# Optimal dosage and effectiveness of imagery practice on athletes’ mental health: a Bayesian multilevel meta-analysis

**DOI:** 10.3389/fpsyg.2025.1618617

**Published:** 2025-08-08

**Authors:** Xuda Zhang, Shiao Zhao, Sanfan Ng, Yiran Liu, Taihe Liang, Chon In Lao, Ziheng Ning

**Affiliations:** Faculty of Health Sciences and Sports, Macao Polytechnic University, Macau, Macao SAR, China

**Keywords:** imagery practice, psychological skills training, athletes, mental health, sports

## Abstract

**Introduction:**

While imagery practice is effective for performance enhancement, its impact on mental health is inconclusive due to mixed findings and heterogeneous athlete populations. This study aims to evaluate the effectiveness and optimal dosage of imagery practice on athletes’ mental health outcomes.

**Methods:**

A total of 24 randomized controlled trials (RCTs), encompassing 1,294 athletes, were synthesized using a Bayesian multilevel meta-analysis in accordance with the PRISMA 2020 guidelines. Among them, 623 were male and 375 were female; the remaining 296 participants were from studies that did not report sex-specific data. The included trials spanned diverse continents (e.g., America, Asia, Europe), covered a wide age range (from adolescents to adults), and involved both individual and team sports (e.g., gymnastics, soccer, swimming). A Bayesian multilevel approach was selected to account for potential clustering within studies and to provide full posterior distributions for effect estimates, allowing for more robust inferences under uncertainty.

**Results:**

Our research indicate that imagery practice may improve athletes’ mental health [μ(SMD): 0.5, 95% CI: 0.34 to 0.56; HDI: 0.22 to 0.89; BF: 17.16], including reducing anxiety levels [μ(SMD): 0.52, 95% CI: 0.11 to 0.96; HDI: 0.11 to 0.96; BF: 2.33], strengthening self-confidence [μ(SMD): 0.62, 95% CI: 0.1 to 1.13; HDI: 0.12 to 1.15; BF: 2.2], and improving self-efficacy [μ(SMD): 1.36, 95% CI: 0.26 to 2.47; HDI: 0.24 to 2.45; BF: 5.38]. In the athlete model, statistically significant effects of imagery practice were found only among tennis players [μ(SMD): 1.16, 95% CI: 0.19 to 2.48; HDI: 0.13 to 2.39; BF: 4.06]. In moderation analysis, a dosage of 45-min sessions once a week for 100 days may be associated with more favorable mental health outcomes.

**Discussion:**

To our knowledge, limited prior meta-analytic research has employed Bayesian multilevel modeling to examine the effects and moderators of imagery practice on mental health outcomes. While the findings suggest that imagery practice may offer psychological benefits for athletes, these effects appear to vary across contexts and athlete populations. Therefore, imagery should be applied with caution, considering individual differences and potential limitations. This study contributes to sport psychology by offering preliminary empirical guidance for tailoring imagery practice to support athletes’ mental health.

**Systematic Review Registration:**

https://www.crd.york.ac.uk/PROSPERO/view/CRD420251050005.

## 1 Introduction

The mental health of athletes is intrinsically linked to their performance and serves as a crucial metric that must not be overlooked in everyday training. Research indicates that student-athletes exhibit a higher susceptibility to severe mental health problems, including anxiety and depression, in comparison to their peers (Grubic et al., 2021). Current data indicate that the prevalence of anxiety or depression among active elite athletes is 34%, and among retired elite athletes, it is 26% (Gouttebarge et al., 2019). The NCAA poll indicates that the majority of student-athletes desire access to educational resources about mental health (Grubic et al., 2021; National Collegiate Athletic Association, 2022). Imagery practice has been increasingly explored as a promising psychological skills training technique for improving mental health and alleviating negative emotional states among athletes in latest reviews (Li et al., 2024; Mesagno and Beckmann, 2017). While some primary studies have explored its psychological effects, to date, no systematic review or meta-analysis has comprehensively evaluated the mental health outcomes of imagery practice in athletic populations. Moreover, there is a lack of evidence regarding its optimal dosage (e.g., frequency, duration) and whether its effectiveness varies according to athlete characteristics such as age and gender. Therefore, this study aims to apply a Bayesian multilevel meta-analysis to synthesize the psychological effects of imagery practice and to examine potential moderators related to dosage and demographic factors.

Imagery denotes a process akin to perception that transpires in the absence of external sensory stimuli (Munzert et al., 2009; Toth et al., 2020). It has convincing theoretical support that explains its mechanisms and guides its practical applications. Mental simulation theory offers strong evidence for the mechanisms behind mental training, explaining both neural activation during motor imagery and its behavioral effects. According to mental simulation theory, motor imagery activates neural substrates involved in actual movement execution, including the premotor cortex, supplementary motor area (SMA), and even the primary motor cortex (M1) (Jeannerod, 2001; Munzert et al., 2009). This internal activation, though covert, engages motor representations that are functionally equivalent to real actions. Crucially, such activation extends beyond motor control to influence autonomic responses, such as heart rate and respiration, which are also implicated in emotional regulation. For instance, imagery-induced activation can produce physiological arousal states that mirror actual performance, enhancing self-perceived readiness and control (Jeannerod, 1994). These embodied responses serve as a pathway through which motor imagery can reduce anxiety, increase confidence, and support broader psychological outcomes, thus linking neural simulation directly to mental health benefits. Additionally, recent studies suggest that mental imagery is not only beneficial for athletic and emotional regulation but also plays a crucial role in basic cognitive functions. For instance, mental imagery has been linked to improved academic performance through its relationship with visuospatial processing (Commodari, 2024), early literacy and numeracy skills (Guarnera et al., 2019), and geometry problem-solving in children (Fastame, 2021). These findings support the idea that imagery practice may serve as a transferable cognitive skill with broad educational relevance beyond elite sport. Nonetheless, despite robust theoretical backing, the practical efficacy of imagery practice remains ambiguous and necessitates additional exploration.

Empirical evidence suggests that imagery practice can produce a range of psychological benefits for athletes. Several studies report that imagery practice is associated with reduced anxiety (Hut et al., 2023; McAlister and Cutler, 2024), enhanced confidence (Fekih et al., 2021; Hidayat et al., 2023), and improved self-efficacy (Cumming et al., 2006; Feltz and Riessinger, 1990). These outcomes are believed to be influenced by athletes’imagery abilities, which may modulate levels of trait confidence and anxiety regulation (Williams and Cumming, 2016). Furthermore, recent research suggests that motor imagery may help mitigate choking under pressure (Harris et al., 2023; Li et al., 2024; Mesagno and Beckmann, 2017), and appears particularly helpful for injured athletes—potentially enhancing confidence and reducing anxiety during recovery (Monsma et al., 2009; Raboin, 2024). There is also preliminary evidence indicating that athletes report high satisfaction with imagery use (Hammond et al., 2012; Raboin, 2024), and that it may be beneficial across both Western and non-Western populations (Chungath et al., 2022).

However, the overall effectiveness of imagery practice on mental health remains subject to debate. Some studies have found little to no benefit, particularly in certain skill-based sports such as gymnastics (Marshall and Gibson, 2017), shooters (Zervas and Kakkos, 1991), football players (Munroe-Chandler et al., 2005), basketball players (Kanthack et al., 2013) and golfers (Martin and Hall, 1995). In some cases, the content of imagery scripts was criticized for lacking relevance or emotional impact. For injured athletes, longer recovery durations have been linked to reduced engagement with imagery and elevated somatic anxiety (Monsma et al., 2009). Additionally, the optimal dose of imagery practice, such as frequency and session length remains poorly understood (McAlister and Cutler, 2024), and may vary depending on individual or contextual factors. Taken together, while the literature indicates promising psychological outcomes for some athletes, findings are inconsistent across athlete populations and sport types. These discrepancies may stem from differences in intervention design, imagery ability, the constructs targeted (e.g., anxiety vs. confidence), or other moderating factors. A comprehensive meta-analysis is therefore warranted to evaluate the overall effectiveness of imagery practice on mental health outcomes and to clarify the roles of key moderators.

Moreover, traditional meta-analyses often ignore multilevel data structures and parameter uncertainty, which a Bayesian framework can address effectively. Therefore, this study aims to investigate the effects of imagery practice on athletes’ mental health, encompassing factors such as anxiety, mood, self-efficacy, and mental toughness, as well as to determine the appropriate dosage of imagery for athletes’ application. Drawing from current yet constrained studies, we suggest two hypotheses: (1) Imagery practice will significantly enhance athletes’ mental health; (2) The effectiveness of imagery practice may vary depending on factors such as gender, age, dosage, and other individual or contextual variables.

## 2 Method

This study was registered with PROSPERO (CRD420251000369) and adhered to the PRISMA reporting guidelines (Haddaway et al., 2022). The completed PRISMA checklist is provided in Supplementary File S0 for reference. A Bayesian meta-analysis was conducted following a systematic review, utilizing Covidence, Python version 3, GRADEprofiler, R version 4.4.3, and GetData Graph Digitizer.

### 2.1 Eligibility criteria

Studies were deemed eligible if they conformed to the PICOS framework criteria:

Population (P): Participants had to be athletes of any age and competitive level, regardless of their physical or mental health status.

Intervention (I): Eligible studies were required to implement imagery practice either as a standalone intervention or as a component of a broader psychological skills training package (PSTP), as long as imagery was clearly included as one of the components.

Comparator (C): The control condition could consist of either no psychological training (no practice) or an alternative PST intervention that did not include imagery practice.

Outcomes (O): Studies needed to report outcomes related to mental health, such as anxiety, depression, stress, or general mental health.

Study Design (S): Only randomized controlled trials (RCTs) were included.

Additional exclusion criteria were also applied:

Unpublished manuscripts, including master’s or doctoral theses, were excluded. Non-original works such as letters, reviews, and editorials were not considered. Studies without extractable data or those in which both the experimental and control groups received different types of imagery practice were also excluded.

### 2.2 Information sources

A comprehensive search method was developed utilizing Medical Subject Headings (MeSH) and free-text search terms to systematically investigate seven English databases, including SportDiscus, PubMed, PsycINFO, Web of Science, MEDLINE, MEDLINE Complete, and CINAHL, on February 24, 2025. The keywords and subject heading were definitively established by discussion among the three authors (SZ, XZ, and ZN). Detailed search strings for each database are provided in Supplementary File S1. A total of 11,621 studies were obtained using the Covidence online tool and ASReview in Python (van de Schoot et al., 2021) for systematic screening.

### 2.3 Study selection and data collection

All abstracts were assessed using the machine learning tool ASReview (van de Schoot et al., 2021). ASReview uses active learning, a type of supervised machine learning, where the model is iteratively trained on reviewer-labeled abstracts as relevant or irrelevant. Based on these labels, the system continually re-ranks the remaining records according to their predicted likelihood of inclusion. This process enables ASReview to prioritize more relevant records for manual review, while reducing the overall screening burden (Holzinger, 2016; Wang et al., 2020). To minimize the risk of omitting eligible studies, we followed a conservative application of the SAFE rule, continuing the ASReview-assisted screening until 200 consecutive records were classified as irrelevant, which suggests a low probability of remaining relevant studies (Boetje and Van De Schoot, 2024). While this approach improves efficiency, we acknowledge the potential for algorithmic bias, particularly in early training stages where inclusion decisions are based on a small number of labeled studies. To mitigate this, the reviewers regularly monitored ASReview’s predictions and ensured a diversity of topics in early training labels.

In the subsequent phase, two reviewers (SZ and XZ) employed the Covidence online tool, as advised by PRISMA, to screen entire texts (Haddaway et al., 2022). Eligible full-texts were assessed using Extraction 1.0 forms, with discrepancies addressed by the third reviewer (Veritas Health Innovation, 2023).

### 2.4 Data items

For each study, the characteristics extracted included authors, publication years, country, intervention, study design, session of imagery practice, sample size, gender, types of athletes, age, training years and outcomes. The outcomes of mental health encompassed worry, self-efficacy, self-criticism, self-concept, perceived difficulty, peaking under pressure, NASA index, mood, mindfulness, mental toughness, mental effort, Hooper index, coping with adversity, confidence and anxiety.

Data were separately extracted by two writers (SZ and XZ) with Covidence, with discrepancies addressed by consultation with the third author (ZN). Data were expressed as mean ± standard deviation (M ± SD). We utilized an online tool named Meta-analysis Accelerator to transform data that was not originally in M ± SD format (Abbas et al., 2024). When data were not presented as exact numbers, Get Data Graph Digitizer (Digitizer, 2020) was used to extract data from graphs.

### 2.5 Risk of bias assessment

The risk of bias for all included studies was assessed independently in accordance with the criteria outlined in the Cochrane Handbook for Systematic Reviews of Interventions (Higgins et al., 2011). Two authors (SZ and XZ) assessed the included studies through the Cochrane risk of bias (ROB2) criteria in RCTs within Covidence. Seven areas of bias were evaluated: (1) Random sequence generation; (2) Allocation concealment; (3) Blinding of participants and personnel; (4) Blinding of outcome assessment; (5) Incomplete outcome data; (6) Selective reporting; and (7) Other bias. The risk of bias was classified as low, unclear, or high. After individual assessments, the authors reached an agreement through discussion. The conclusive data were recorded in an Excel template and subsequently input into R software to produce risk of bias summary graphs using the robvis package (McGuinness and Higgins, 2021). Studies with more than two but fewer than four categories classified as unclear risk were deemed to have moderate overall risk.

### 2.6 Publication bias

To evaluate potential publication bias, we employed the PublicationBias R package (Braginsky et al., 2019; Mathur and VanderWeele, 2020), which adopts a sensitivity analysis approach that does not rely on the assumption of funnel plot symmetry. This method estimates the magnitude of publication bias necessary to reduce the observed effect size or its confidence interval to a predefined threshold.

Assuming that statistically significant positive findings were preferentially published (i.e., favor_positive = TRUE), we generated a significance funnel plot to visually differentiate between affirmative results (significant and positive) and non-affirmative ones (either nonsignificant or negative). We also derived the worst-case estimate, calculated solely from non-affirmative studies, as a reference point to assess the robustness of the main findings. In addition, we computed the s-value, which represents the smallest selection ratio (*η*) required to reduce the effect size to zero. An s-value of “not possible” suggests that no plausible publication bias could fully explain the observed effect.

To complement this modern approach, we also conducted traditional bias assessments using the metafor package. These included a conventional funnel plot, Egger’s test, and, when Egger’s test indicated significant (*p* < 0.05), we applied the trim-and-fill method. These Frequentist tools provided a secondary line of evidence regarding potential publication bias.

### 2.7 Certainty in evidence

We assessed the quality of evidence using the GRADE approach, following the guidance provided by Ryan (2016). According to the GRADE framework, the quality of evidence may be downgraded based on five domains:

1. Risk of bias: no downgrading was applied when all or the majority (i.e., at least two-thirds) of the information came from studies assessed as having low risk of bias. When the majority of the evidence came from studies with unclear or moderate risk of bias, the quality was downgraded by one level. If the majority of the information originated from studies at high risk of bias, the evidence was downgraded by two levels.
2. Inconsistency: inconsistency was downgraded by one level when *I*^2^ was between 50 and 75%, and by two levels when *I*^2^ exceeded 75%.
3. Imprecision: The imprecision criterion is met if the 95% confidence interval (CI) crosses the equivalence line: 0 for standardized mean differences. Additionally, imprecision is considered when the total number of events or cases across all included studies does not meet the Optimal Information Size (OIS), with thresholds of less than 400 for continuous variables. Meeting one of these criteria warrants downgrading the evidence quality by one level, while meeting both criteria warrants downgrading by two levels.
4. Indirectness: when the evidence does not directly address the research question in terms of population, intervention, comparator, or outcome.
5. Publication bias: we downgraded the quality of evidence by one level when both of the following conditions were met: (1) all included randomized controlled trials were of small sample size (approximately 30 participants per group), and (2) the studies were sponsored by industry or manufacturers.

The GRADE methodology categorizes the quality of evidence as high, moderate, low, or very low.

### 2.8 Statistical analysis

Bayesian mixed-effects models, executed via the brms package (Bürkner, 2018) in R 4.4.3, were employed to analyze variation in effect sizes. Given the inclusion of multiple outcomes per study, a two-level model was implemented: level 1 (effect sizes within studies), level 2 (study-level variation). The Bayesian framework is particularly appropriate for meta-analyses involving a small number of studies, as it allows for the evaluation of both null and alternative hypotheses and yields comprehensive insights into the credibility and probability distribution of parameter estimates (Harrer et al., 2021; Kruschke and Liddell, 2018; Röver, 2020). Random intercepts were specified at both levels to account for clustering and study-specific variability. We constructed models assuming a normal distribution and included random effects for within-study ID and between-study ID to account for heterogeneity in each outcome, utilizing two formulas:


$$I_{\text{within}}^{2}=\frac{\tau_{\text{withID}}^{2}}{\text{total}\_\mathrm{Var}\text{iance}}\times 100$$

$$I_{\text{between}}^{2}=\frac{\tau_{\text{betweenID}}^{2}}{\text{total}\_\mathrm{Var}\text{iance}}\times 100$$

Weakly informative priors (mean = 0, standard deviation = 1) were utilized for the random effects (Harrer et al., 2021). Mental health outcomes were examined and categorized according to the imaging tactics employed (imagery alone or imagery practice combined with additional psychological skills training), athlete categories, and mental health results. Four models were conducted as follow:

1. Null model to estimate overall effect sizes. We fitted models on the full dataset for mental health.
2. Imagery model: We classify imagery practice based on its unique characteristics. The first category encompasses imagery practice that is not combined with other psychological skills training, whereas the second category comprises psychological skills training packages (PSTP) that include imagery practice. Secondly, a comparison will be conducted between these two modalities of imagery practice and other psychological skills training, as well as the lack of psychological training (no practice).
3. Mental health model: We categorize the derived mental health results according to their characteristics.
4. Athlete model: Athletes are classified based on the sport in which they engage.

Each model was estimated utilizing four Markov chains, with each chain executing 10,000 iterations. We exclusively utilized the Rhat statistic to assess the convergence of the Markov chains. Rhat evaluates the ratio of between-chain to within-chain variation, with values approaching 1 indicating adequate convergence. This measure offers a dependable evaluation of convergence, confirming that chains with Rhat values close to 1 have comprehensively examined the posterior distribution, thus improving the dependability of model inference. Bayes Factors (BFs) (Makowski et al., 2019) were calculated using the reciprocal of the Savage-Dickey density ratio, implemented through the bayesfactor_parameters function in the bayestestR package. Furthermore, Bayes Factors (BF) are commonly interpreted on a graded scale, where BF > 3 represents moderate evidence and BF > 10 indicates strong evidence in favor of the effect (Kass and Raftery, 1995). In complicated hierarchical models, the 95% high-density interval (HDI) (Kruschke and Liddell, 2018) provides a direct and assumption-free depiction of the most likely posterior values. This contrasts with *p*-values and confidence intervals, which depend on further assumptions.

### 2.9 Moderation analysis

In this study, moderation analysis was conducted using the brms package in R, including five moderator variables: age, gender, training experience (in years), competitive level, sport type (team or individual), and three moderators included imagery practice duration (in days), weekly practice frequency (sessions per week), and intensity (minutes per session) were examined to determine the optimal dosage of imagery practice for improving mental health. The study was performed using a Bayesian framework, facilitating the calculation of effect sizes while considering measurement error and the hierarchical structure of the data.

To investigate the effects of different moderators on model performance, we first constructed a null model without any moderator variables. Subsequently, we developed separate models, each incorporating a single moderator. The Bayesian R-squared statistic, computed using the bayes_R2 function from the brms package, was employed to estimate the proportion of variance explained by each model. In order to compare the explanatory power of models with and without moderators, we visualized the distribution of *R*-squared values using density plots. This approach enables a more intuitive understanding of how the inclusion of moderators contributes to model improvement and facilitates direct comparisons with the null model.

## 3 Results

The results include six parts, namely study selection, the characteristic of included studies, quality assessment, meta-analysis with four models, moderation analysis, quality grade and publication bias.

### 3.1 Study selection

**Figure 1:**
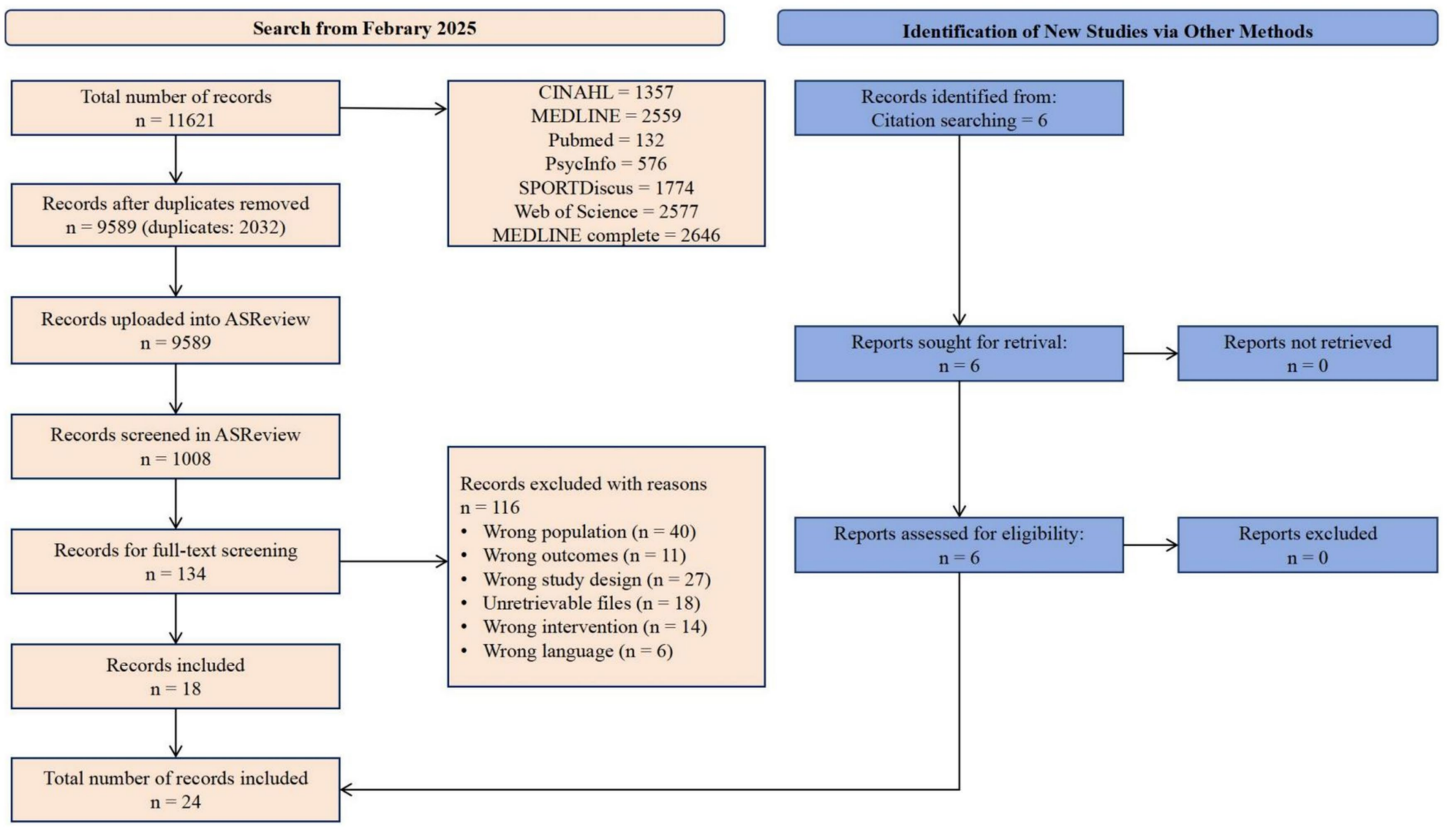
PRISMA flow chart for the identification of the included studies.

Figure 1 illustrates the flow chart. A total of 11,621 articles were extracted from seven databases and put into Endnote software for the purpose of eliminating duplicates. Endnote automatically identified and eliminated 2,032 studies as duplicates. A total of 9,589 papers were loaded into the ASReview tool for preliminary screening, encompassing the evaluation of titles and abstracts. Utilizing machine learning models, 1,008 articles were evaluated, resulting in 134 articles being included for full-text screening. Following full-text screening, 116 studies were removed for various reasons, whereas 18 articles were incorporated into this meta-analysis. Six additional studies were incorporated through the citation searching strategy. A total of 24 studies were incorporated into the meta-analysis.

### 3.2 Characteristic of included studies

The specific characteristics of each study included are available in Supplementary File S2. Twenty-four studies including 1,294 athletes, consisting of 375 females and 623 males, were considered, with 296 participants lacking gender data (Alwan et al., 2013; Kanthack et al., 2013; Mguidich et al., 2024). The included studies involved a diverse range of athlete populations, including dancer (*n* = 1), gymnast (*n* = 1), golfer (*n* = 1), tennis players (*n* = 3), volleyball player (*n* = 1), basketball players (*n* = 4), ice hockey athlete (*n* = 1), swimmers (*n* = 2), CrossFit athlete (*n* = 1), karateka (*n* = 1), soccer players (*n* = 3), badminton athlete (*n* = 1), runner (*n* = 1), weight lifter (*n* = 1), and rugby player (*n* = 1).

With the exception of three research conducted as randomized crossover trials (Page et al., 1999; Rumeau et al., 2023; Taylor and Shaw, 2002), all remaining investigations were randomized parallel trials. Six studies used a combination of imagery practice and other psychological training as intervention methods (25%). Five studies were from Asia (20.8%). Nine studies were from Europe (37.5%). Seven studies were from America (29%). Two studies were from Oceania (8%) and one study were from Africa (4%). Three studies did not provide age information (Alwan et al., 2013; Kanthack et al., 2013; Mguidich et al., 2024).

### 3.3 Quality assessment

**Figure 2:**
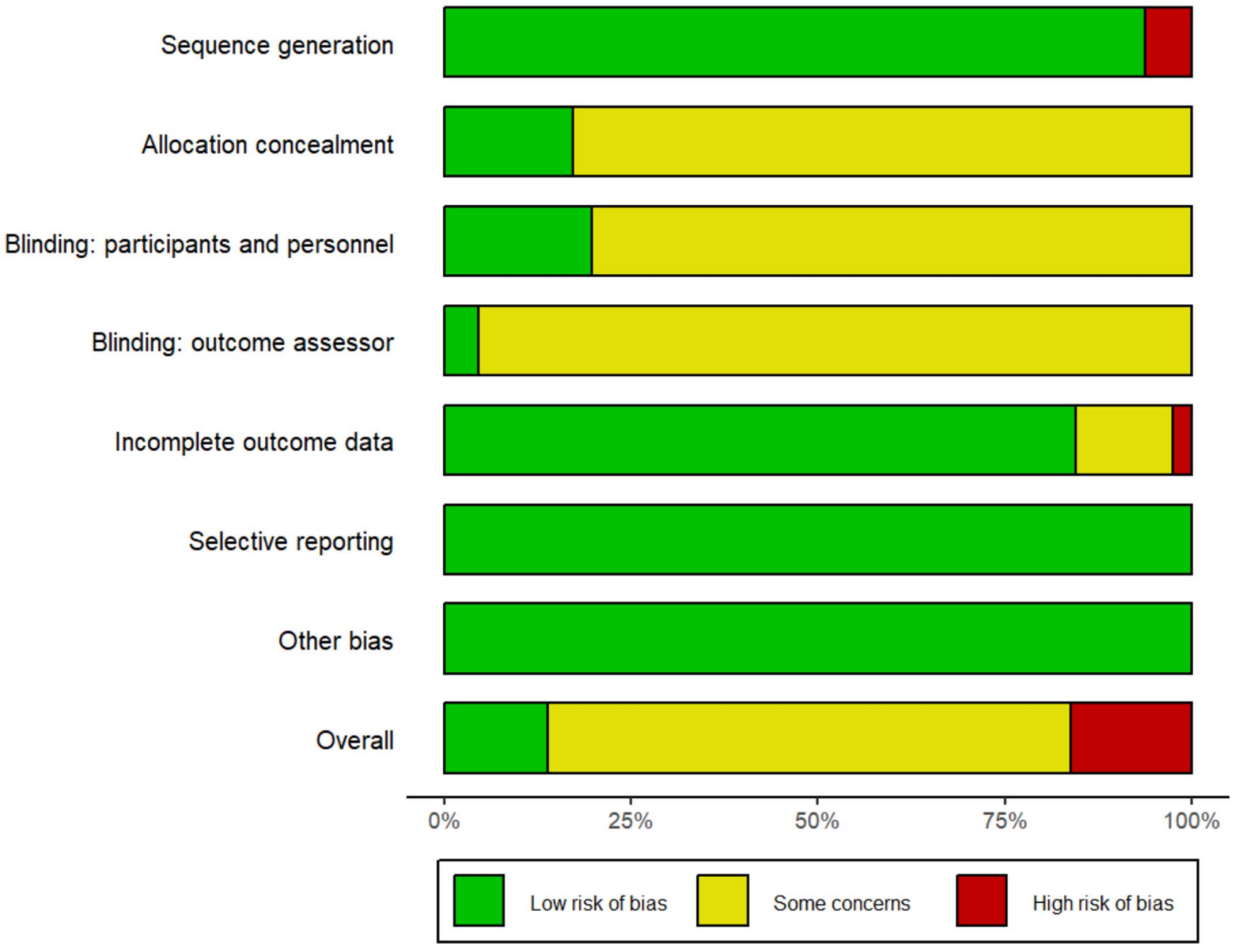
Risk of bias summary.

The risk-of-bias summary is illustrated in Figure 2, while the detailed risk-of-bias graph for each study is available in Supplementary File S3. Some studies were classified as high risk (8%) due to the erroneous application of randomization methods, such as block randomization (Marshall and Gibson, 2017; Noh et al., 2007). Over 80% of studies failed to report the implementation of blinding, resulting in an assessment of unclear risk. Due to incomplete data, two studies were classified as having an unclear risk (Alwan et al., 2013; McAlister and Cutler, 2024) and one study was marked as high risk (Hut et al., 2023). No articles have been marked as unclear or high risk in the areas of selective reporting and other biases. More than 50 % of the research were classified as having an unclear risk, hence diminishing the robustness of the data outcomes.

### 3.4 The results of meta-analysis

The meta-analysis is divided into four sections. The first section included the Null model, followed by the imagery model, mental health model and athlete model. The detailed results in each model are illustrated in Supplementary File S4. The convergence of the Markov chain in all four models was accurately evaluated using the Rhat parameter value in the results. The Rhat value across all outcomes was roughly 1.0. Therefore, we did not include the findings on Markov chain convergence in the article.

#### 3.4.1 Null model

**Figure 3:**
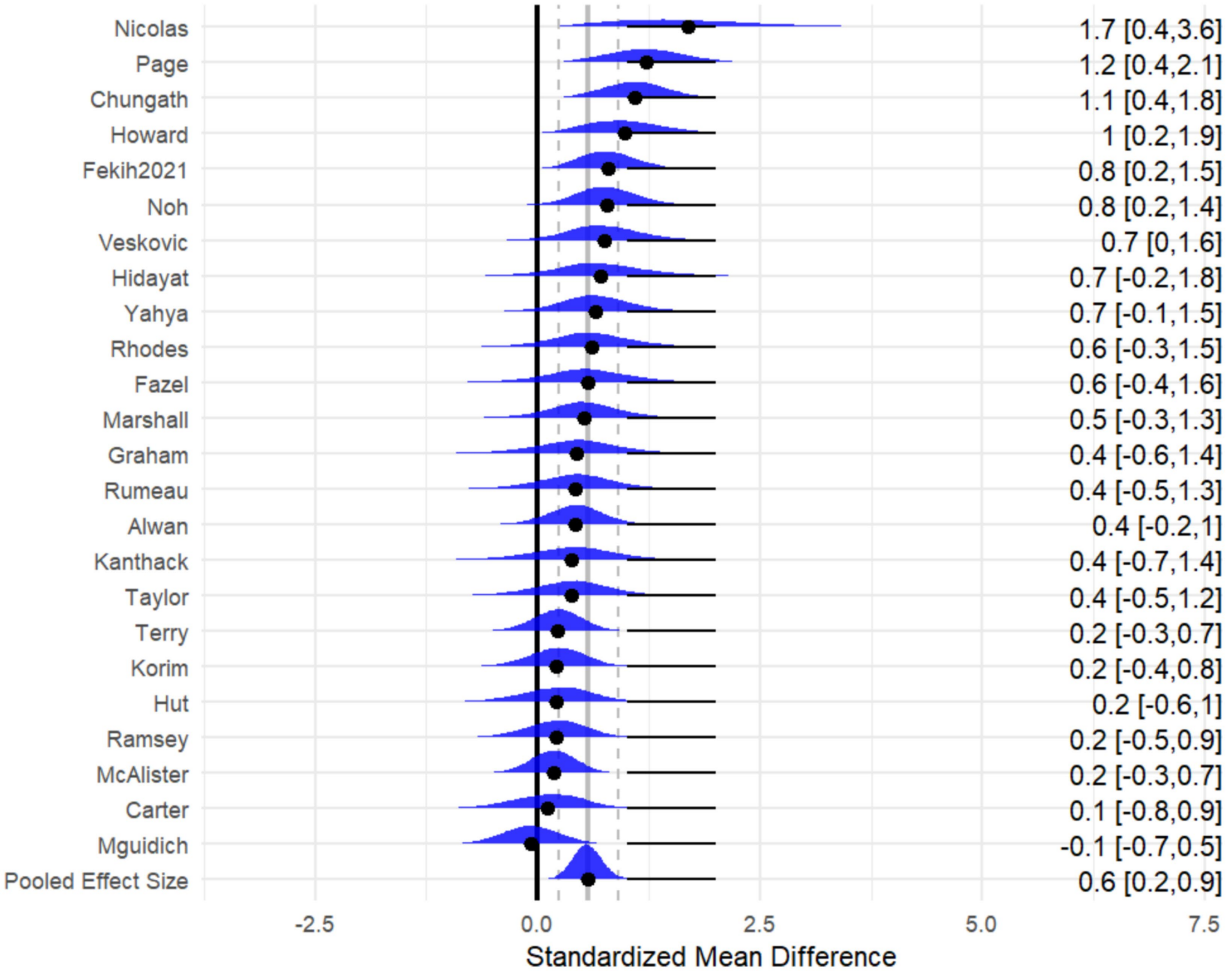
The forest plot in null model.

In the null model, the overall effect size was calculated for mental health. Twenty-four studies with 1,294 athletes were included in the null model. The forest plot is illustrated in Figure 3. The Bayesian meta-analysis showed a statistically significant effect [*μ*(SMD): 0.5, 95% CI: 0.34 to 0.56; HDI: 0.22 to 0.89; BF: 17.16], with low between-study heterogeneity and moderate within-study heterogeneity [within *I*^2^: 71.37%, between *I*^2^: 28.63%].

**Figure 4:**
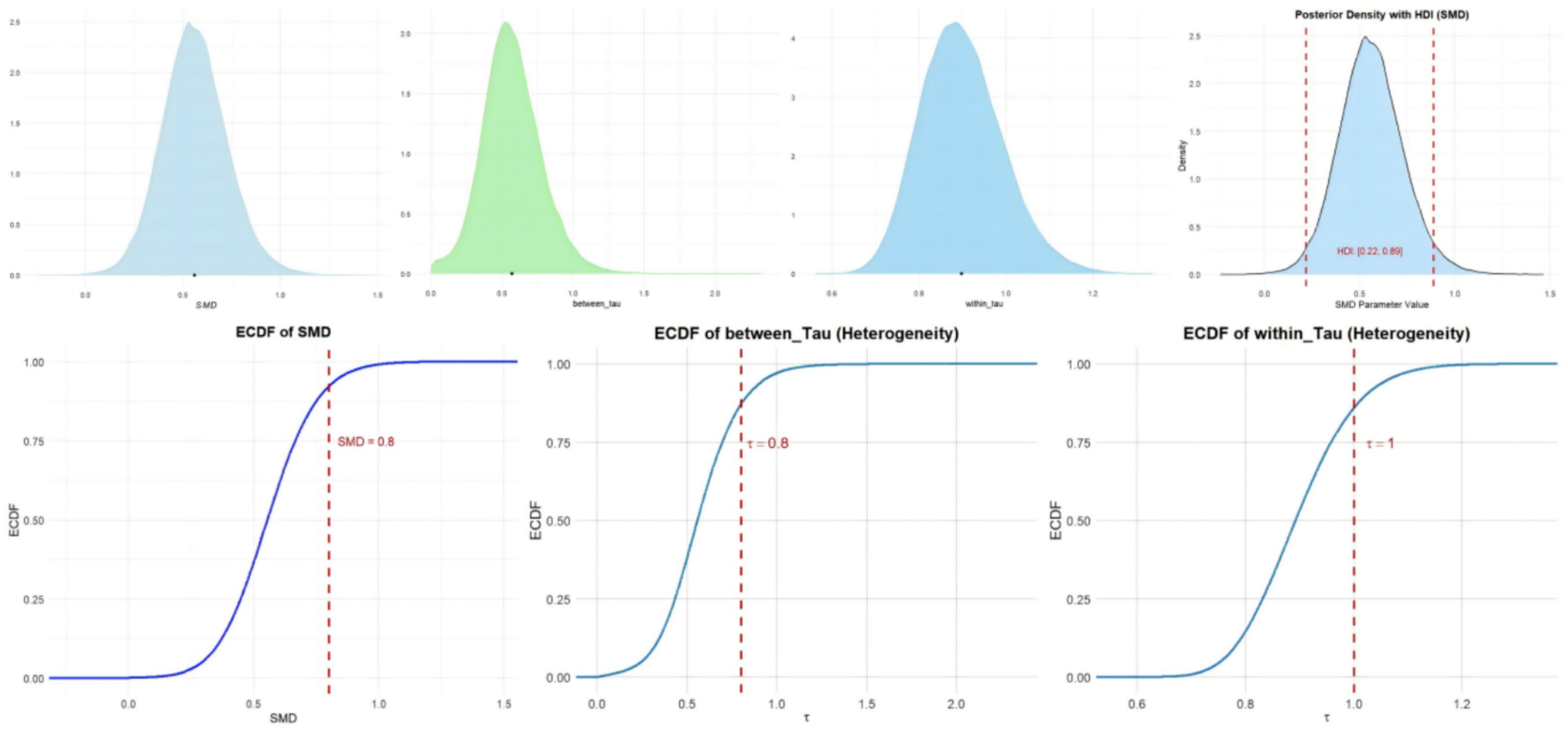
The cumulative probability, HDI distribution and posterior density distribution (SMD, τ_within_ and τ_between_) in athletic performance.

Imagery practice can effectively improve athletes’ mental health. Figure 4 shows the cumulative probability and posterior density distribution of SMD, *τ*_within_ and τ_between_ in athletic performance. As illustrated in Figure 4, the posterior density distributions of τ_between and τ_within indicate substantial heterogeneity between studies (τ ≈ 0.8). Additionally, the posterior distribution of the standardized mean difference (SMD) suggests a moderately significant effect (SMD ≈ 0.6).

#### 3.4.2 Mental health model

**Figure 5:**
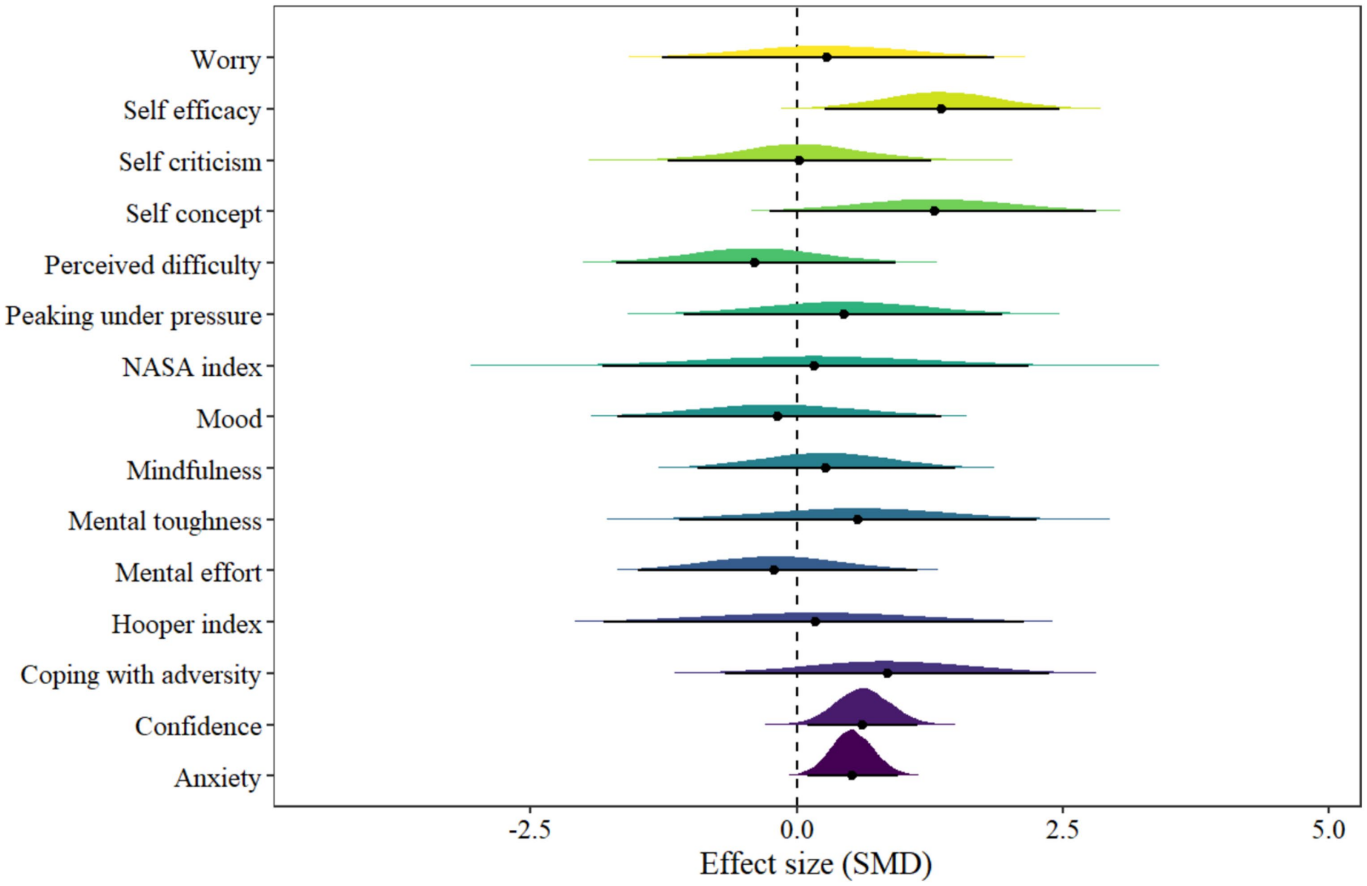
The forest plot in mental health model.

The mental health model presents 15 outcomes on various mental health markers, as illustrated in Figure 5 of the forest plot. However, only three indicators of mental health showed statistical significance, including anxiety [μ(SMD): 0.52, 95% CI: 0.11 to 0.96; HDI: 0.11 to 0.96; BF: 2.33], confidence [μ(SMD): 0.62, 95% CI: 0.1 to 1.13; HDI: 0.12 to 1.15; BF: 2.2] and self-efficacy [μ(SMD): 1.36, 95% CI: 0.26 to 2.47; HDI: 0.24 to 2.45; BF: 5.38], with low within-study and between-study heterogeneity [within *I*^2^: 17.11%, between *I*^2^: 18.67%].

#### 3.4.3 Imagery model

**Figure 6:**
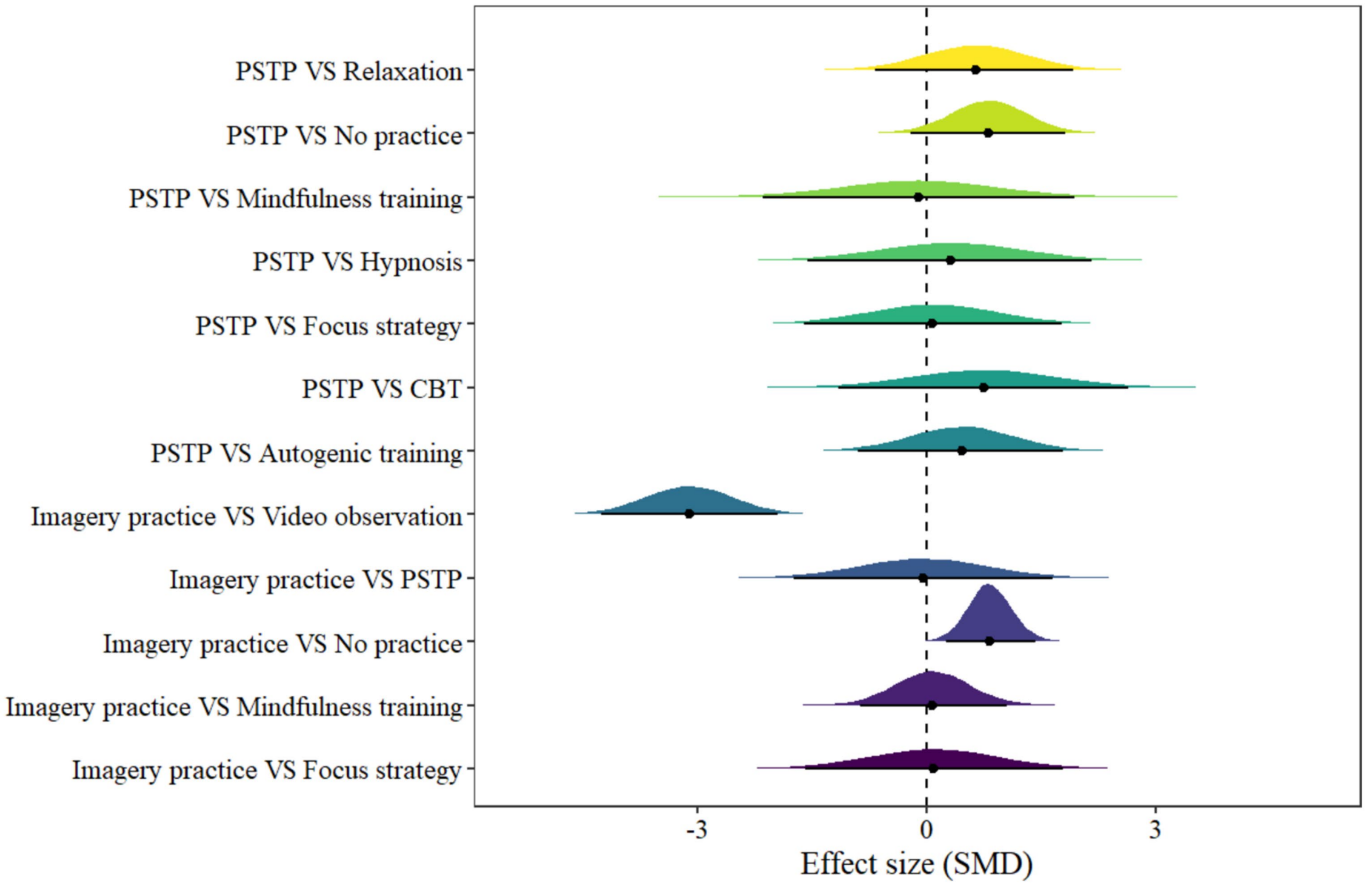
The forest plot in imagery model.

In imagery model, 12 pairs of comparisons were included. The forest plot is illustrated in Figure 6. Only two pair of comparisons showed statistical significance, including Imagery practice VS No practice [μ(SMD): 0.84, 95% CI: 0.26 to 1.43; HDI: 0.25 to 1.42; BF: 9.13] and Imagery practice VS Video observation [μ(SMD): –3.1, 95% CI: −4.26 to −1.95; HDI: −4.24 to −1.93; BF: 4680], with low within-study heterogeneity and high between-study heterogeneity [within *I*^2^: 5.62%, between *I*^2^: 71.33%].

#### 3.4.4 Athlete model

**Figure 7:**
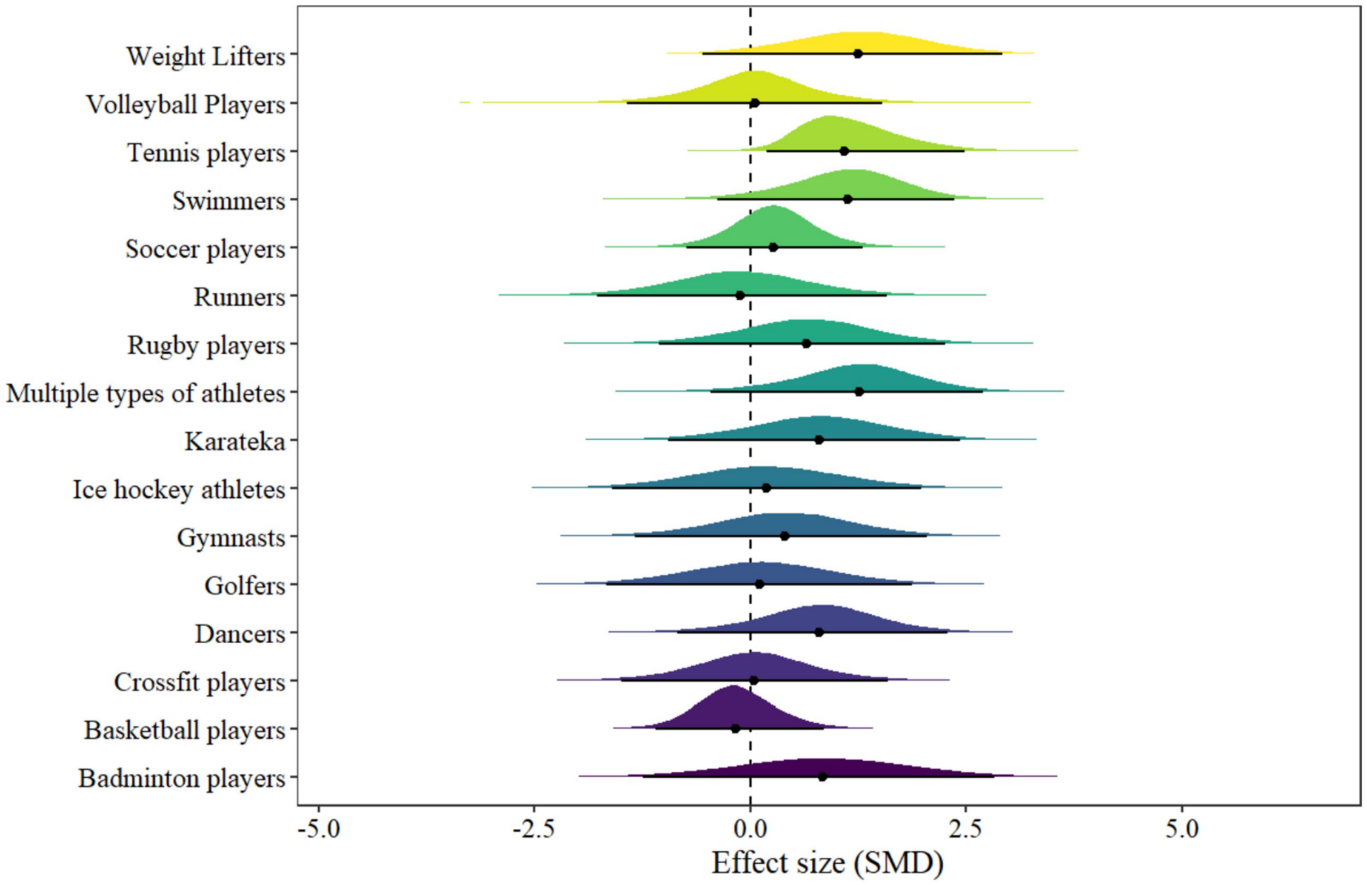
The forest plot in athlete model.

In athlete model, 16 types of athlete results were included. The forest plot is presented in Figure 7. Only one type of athlete showed statistical significance, including tennis players [μ(SMD): 1.16, 95% CI: 0.19 to 2.48; HDI: 0.13 to 2.39; BF: 4.06], with low within-study and between-study heterogeneity [within *I*^2^: 14.42%, between *I*^2^: 33.44%].

### 3.5 Moderation analysis

Five moderators regarding athletes characteristics including age, gender, training experience (in years), competitive level, sport type (team or individual), and three moderators concerning imagery dose encompassing imagery practice duration (in days), frequency (sessions per week) and intensity (minutes per session) were included in the moderation analysis. Regarding the moderation analysis of population characteristics, the charts are illustrated in the Supplementary File S5. The detailed results of moderation analysis by population characteristics is presented in Table 1. The results in the gender model show that imagery practice was less effective among female athletes than males (estimates: −0.01, −0.01 to −0.001). In the model of competitive level, imagery practice is more effective for elite athletes (estimates: 0.95, 0.24 to 1.67). In the model of sport type (team or individual), imagery practice was more effective on individual sports (estimates: 0.69, 0.4 to 0.99).

**Table 1:** The detailed results of moderation analysis by population characteristics.

	Moderator	Estimate	l-95% CI	u-95%CI	*R* ^2^	l-95% CI	u-95%CI
1	Gender	−0.01	−0.01	−0.001	0.85	0.77	0.91
2	Age	−0.04	−0.08	0.01	0.8	0.69	0.89
3	Training years	−0.08	−0.21	0.05	0.79	0.5	0.94
6	Competitive level (Amateur)	−0.5	−1.2	0.18	0.75	0.64	0.85
7	Competitive level (Elite)	0.95	0.24	1.67	0.75	0.64	0.85
9	TI (Individual sports)	0.69	0.4	0.99	0.85	0.78	0.91
10	TI (Team sports)	−0.61	−1.04	−0.19	0.85	0.78	0.91
11	TI (Mix)	0.75	−0.13	1.62	0.85	0.78	0.91
14	Base	0.47	0.25	0.69	0.84	0.77	0.9

TI: team or individual sports.

The optimal modality appeared to be moderated by gender and sport type (team or individual). Compared with base model (*R*^2^ = 0.84), *R*^2^ was higher for gender model and sport type model (*R*^2^ = 0.85). *R*^2^ in training experience model (in years) (*R*^2^ = 0.79) and competitive level model (*R*^2^ = 0.75) is lower than base model. The regression plots and *R*^2^ density plot is presented in Supplementary File S6.

**Figure 8:**
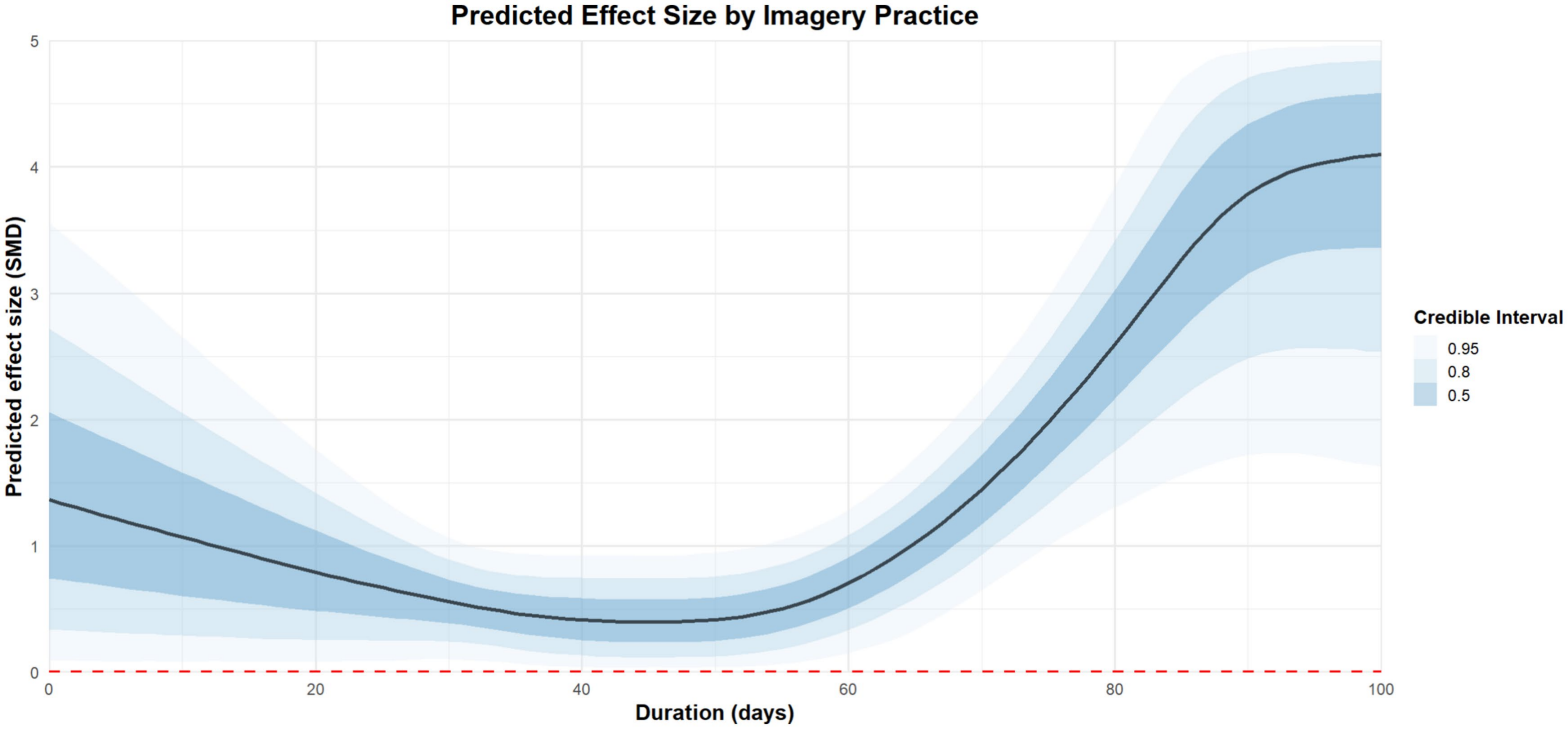
The moderation analysis by duration (days).

Three moderators concerning imagery dosage were incorporated into the moderation analysis. Regarding the duration of imagery practice, 100 days of imagery practice [coefficient estimates: 5.52, 95%CI: 2.77 to 8.11] is more effective than 80 days [coefficient estimates: 2.59, 95%CI: 1.52 to 3.64], 50 days of practice [coefficient estimates: 0.38, 95%CI: −0.08 to 0.85] and 20 days [coefficient estimates: 0.75, 95%CI: −0.08 to 1.59]. The regression plot is presented in Figure 8.

**Figure 9:**
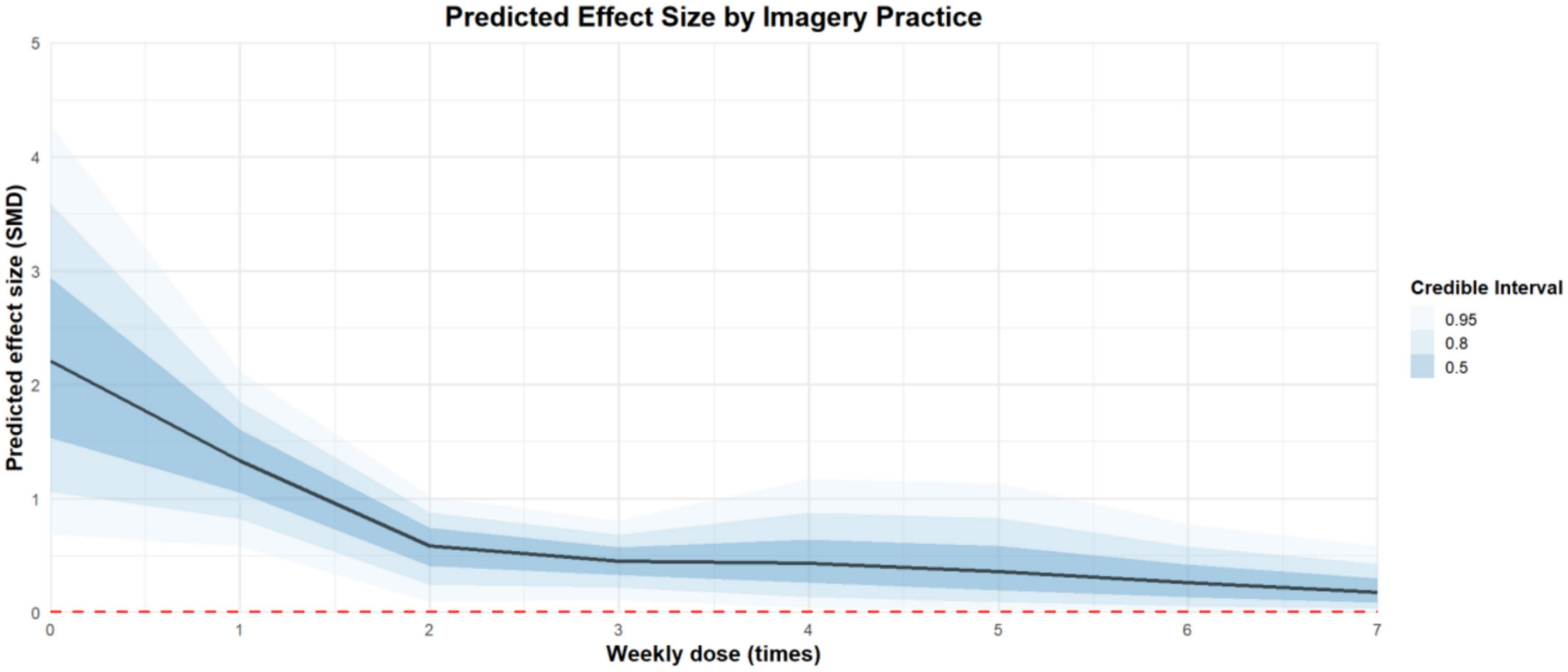
The moderation analysis by weekly dose (times).

Regarding the weekly dose of imagery practice, the effect of practicing one time a week [coefficient estimates: 1.33, 95%CI: 0.69 to 1.99] is better than practicing three times a week [coefficient estimates: 0.45, 95%CI: 0.15 to 0.74] or seven times a week [coefficient estimates: 0.05, 95%CI: −0.35 to 0.44]. The regression plot is presented in Figure 9.

**Figure 10:**
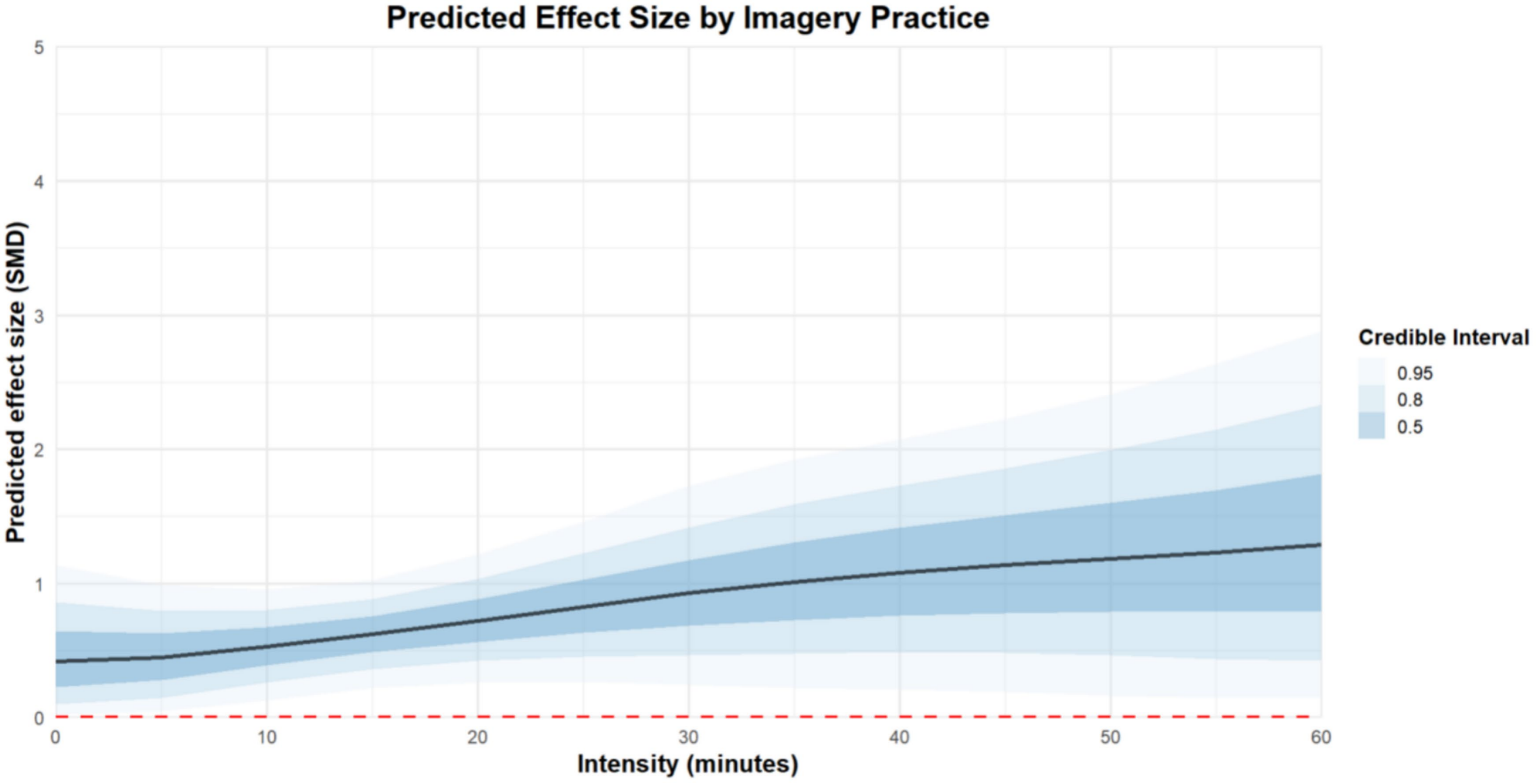
The moderation analysis by intensity (minutes).

Regarding the intensity of imagery practice, practicing for 45 min at a time [coefficient estimates: 1.12, 95%CI: 0.2 to 2.04] is more effective than practicing for 10 [coefficient estimates: 0.53, 95%CI: 0.18 to 0.88], 20 [coefficient estimates: 0.73, 95%CI: 0.33 to 0.12], 30 [coefficient estimates: 0.93, 95%CI: 0.33 to 1.57], and 60 min [coefficient estimates: 1.22, 95%CI: −0.18 to 2.6]. The regression plot is presented in Figure 10.

Thus, the optimal modality appeared to be moderated by duration. Compared with base model (*R*^2^ = 0.84), *R*^2^ was higher for duration model (*R*^2^ = 0.91) and intensity model (*R*^2^ = 0.89). *R*^2^ in weekly model (*R*^2^ = 0.77) is lower than base model. The *R*^2^ density plot is presented in Supplementary File S6. The detailed results of moderation analysis by imagery dose is presented in Table 2.

**Table 2:** The detailed results of moderation analysis by imagery dose.

	Moderator	Type	Estimate	l-95% CI	u-95%CI	R^2^	l-95% CI	u-95%CI
1	Duration	20 days	0.75	−0.08	1.59	0.91	0.86	0.95
2	Duration	50 days	0.38	−0.08	0.85	0.91	0.86	0.95
3	Duration	80 days	2.59	1.52	3.64	0.91	0.86	0.95
4	Duration	100 days	5.52	2.77	8.11	0.91	0.86	0.95
5	Weekly dose	One time a week	1.33	0.69	1.99	0.77	0.62	0.87
6	Weekly dose	Three times a week	0.45	0.15	0.74	0.77	0.62	0.87
7	Weekly dose	Seven times a week	0.05	−0.35	0.44	0.77	0.62	0.87
8	Intensity	10 min	0.53	0.18	0.88	0.89	0.82	0.94
9	Intensity	20 min	0.73	0.33	1.12	0.89	0.82	0.94
10	Intensity	30 min	0.93	0.33	1.57	0.89	0.82	0.94
11	Intensity	45 min	1.12	0.2	2.04	0.89	0.82	0.94
12	Intensity	60 min	1.22	−0.18	2.6	0.89	0.82	0.94
13	Base	NA	0.47	0.25	0.69	0.84	0.77	0.90

### 3.6 Quality grade in each outcome

**Figure 11:**
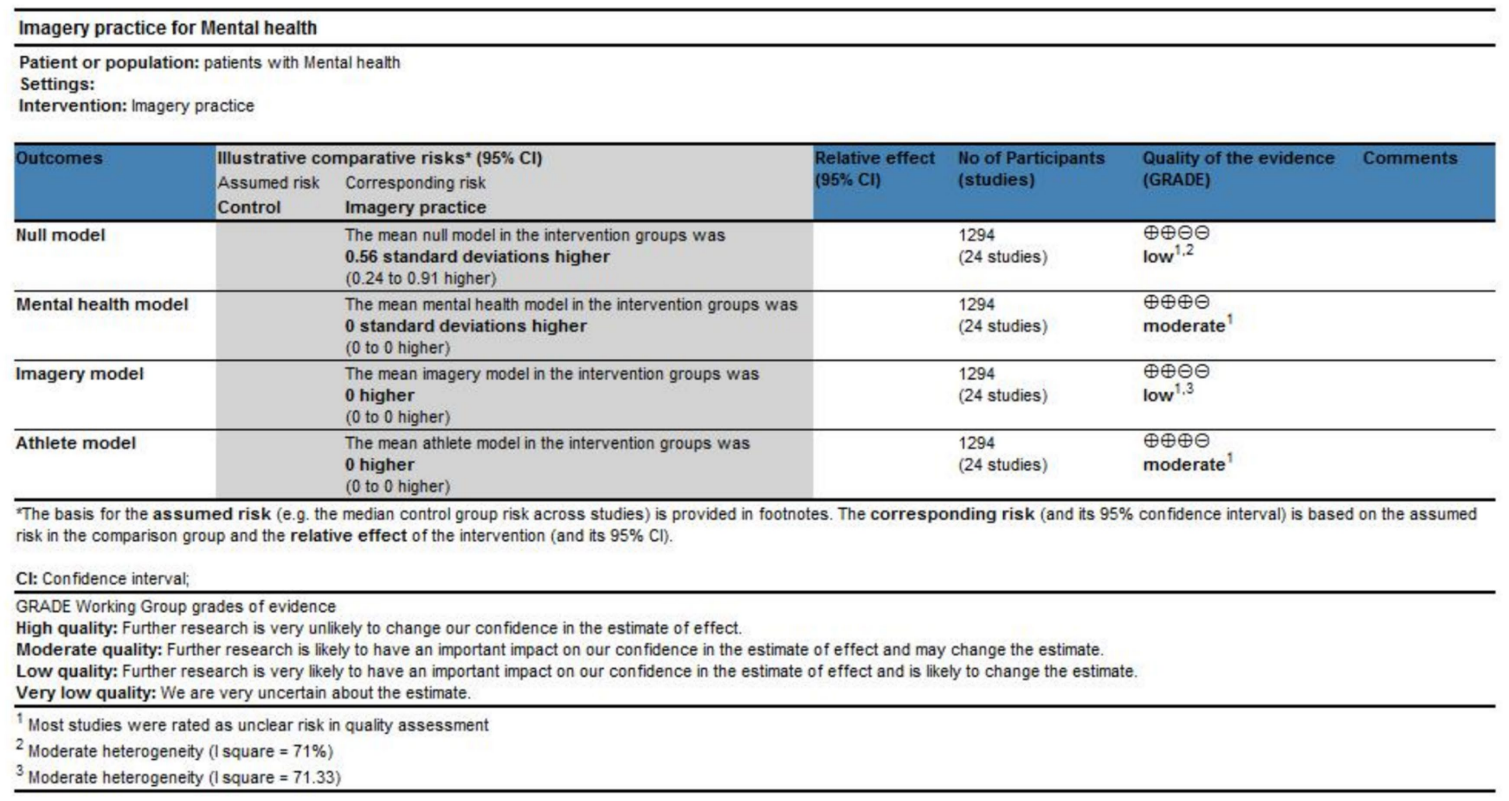
Quality grade of mental health.

The quality grade in mental health was based on the sample size, results of meta-analysis, and quality assessment to assess the risk of bias, inconsistency of results, indirectness, and imprecision of effect estimates. The results showed that the quality grade of the null model was low due to potential risk of bias and considerable heterogeneity. However, because the level of heterogeneity was substantially reduced in the mental health model and the athlete model, their quality grades were rated as moderate. In contrast, the quality grade of the imagery model remained low. The plot is illustrated in Figure 11.

### 3.7 Publication bias

**Figure 12:**
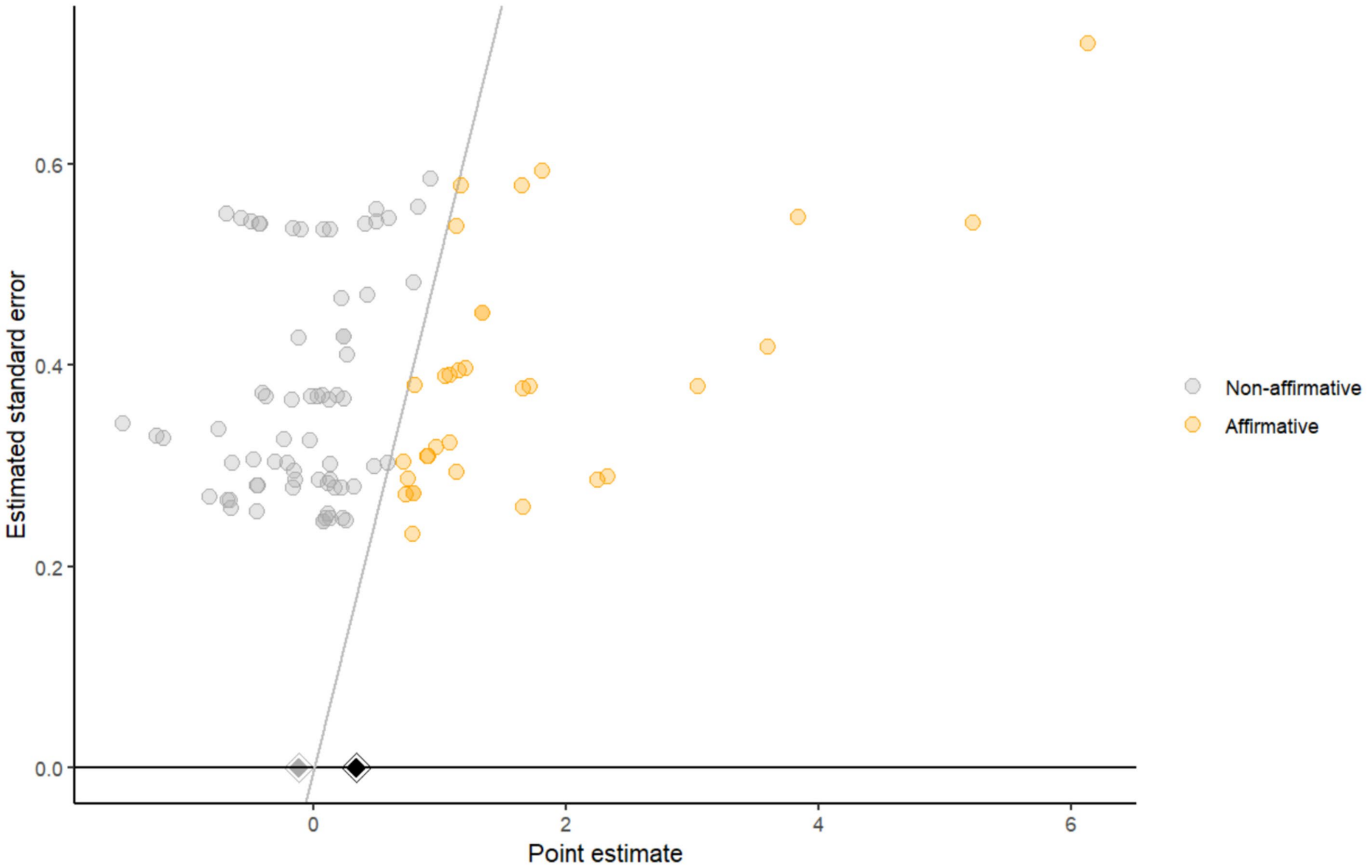
The funnel plot by Publicationbias package.

In the funnel plot generated using the PublicationBias package, studies were categorized as either affirmative (i.e., statistically significant and in the expected direction) or non-affirmative. A disproportionate concentration of affirmative studies was observed, particularly among those with higher standard errors (i.e., smaller sample sizes), suggesting the possibility of selective reporting driven by statistical significance rather than random variation. The funnel plot is illustrated in Figure 12.

To quantify the degree of bias required to attenuate the observed effect size to zero, we calculated the *s*-value. The analysis yielded an *s*-value of 8.86, with a 95% confidence interval lower bound of 1.96. This indicates that affirmative studies would need to be approximately 8.86 times more likely to be published than non-affirmative studies for the overall effect to be explained away. As the lower bound exceeds 1, the observed effect appears relatively robust and unlikely to be fully attributable to publication bias.

**Figure 13:**
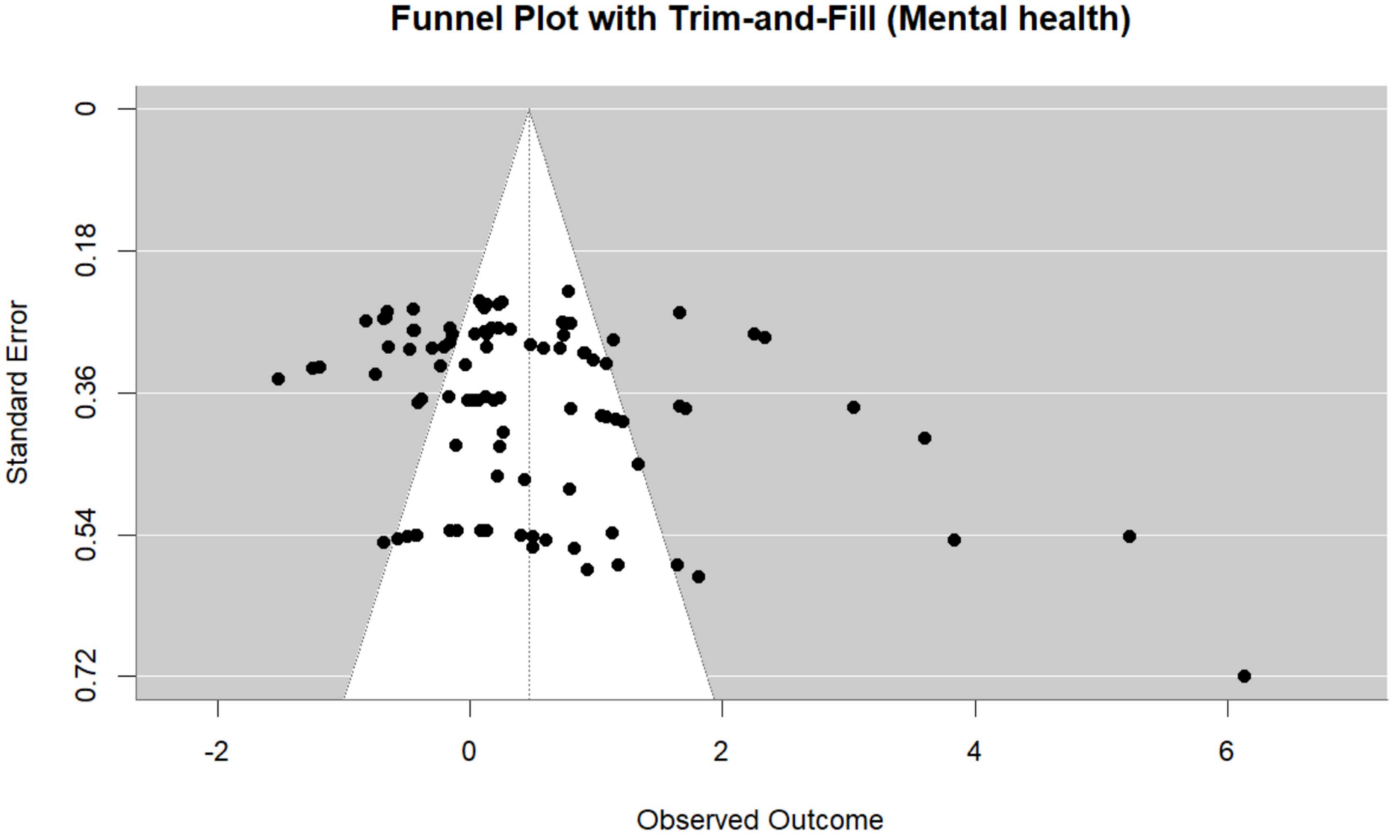
The trim-and-fill funnel plot by Metafor package.

Additionally, a traditional funnel plot generated using the metafor package revealed asymmetry, with an overrepresentation of studies on the right-hand side of the plot. Egger’s test further confirmed this asymmetry (*p* = 0.0005), indicating potential small-study effects. However, the Trim-and-Fill procedure did not identify any missing studies on the left side of the plot (estimated number = 0, SE = 4.92), suggesting that the observed asymmetry may not reflect a simple pattern of missing negative or non-significant studies. Taken together, while the Trim-and-Fill method did not impute missing studies, the results from the significance funnel plot and Egger’s test indicate that some degree of publication bias is likely present, albeit not necessarily in a form detectable by symmetry-based approaches. The Trim-and-Fill funnel plot is illustrated in Figure 13.

## 4 Discussion

To our knowledge, this is the first Bayesian multilevel meta-analysis investigating the impact of imagery practice on athletes’ mental health. This systematic review and meta-analysis consolidate information about the impact of (1) imagery practice alone or imagery in conjunction with other psychological skills training (PST) on mental health, and (2) the dosage of imagery on athletes’ mental health.

### 4.1 Summary of finding

This meta-analysis demonstrates that imagery practice may improve athletes’ mental health, particularly in reducing anxiety and enhancing self-efficacy and confidence. The effect is more pronounced among male and individual sport athletes, with tennis players showing the strongest improvement. Notably, the combination of imagery and other psychological skills training did not yield additional benefits. A dosage of 45 min per week over 100 days may be associated with more favorable outcomes. However, given the limited number of included studies (only 24 RCTs), this study should be considered preliminary. The findings must be interpreted with caution, and more high-quality empirical research is needed to provide stronger evidence.

### 4.2 The effect of imagery practice on mental health

The findings indicate that imagery practice may positively impact athletes’ mental health, leading to decreased anxiety, heightened self-confidence, and improved self-efficacy, particularly among tennis players. Certain investigations furnish corroborative evidence for this finding (Dhiman and Bedi, 2010; Fekih et al., 2021; Lee Howard and Reardon, 1986; Page et al., 1999; Zandi and Masomi, 2010; Afzali and MolaNorouzi, 2023). Some scholars have revealed potential mechanisms underlying the effectiveness of imagery practice, indicating that improvements in cognitive ability may lead to reduced state anxiety (Li et al., 2024). Positive imagery is considered an important predictor of cognitive anxiety, as a confident mindset plays a crucial role in effectively alleviating competition-related stress among young individuals (Strachan and Munroe-Chandler, 2006). However, during the implementation of imagery practice, researchers should pay attention to whether athletes have prior experience with imagery practice. Athletes lacking imagery practice experience may exhibit reduced imagery vividness and controllability (Page et al., 1999). Therefore, while imagery practice may be a promising tool for supporting athletes’mental health, its application should be individualized, and future studies are encouraged to compare it systematically with other psychological strategies to determine context-specific effectiveness.

Moreover, the findings indicate that imagery practice can augment athletes’ confidence and self-efficacy. Certain studies have proposed that Motivational General-Mastery (MG-M) imagery is an advisable type of imagery practice for enhancing athletes’ self-efficacy, with positive emotions playing a substantial role in the association between MG-M imagery utilization and self-efficacy (Hammond et al., 2012; Wirbiezcas, 2019). Athletes with higher levels of self-efficacy tend to use imagery more frequently and have better imagery abilities (Volgemute et al., 2021), and there is a significant positive correlation between athletes’ imagery ability, self-efficacy, and athletic performance (Volgemute et al., 2024). Additionally, Di corrado et al. (2025) have suggested that there is also a strong positive correlation between dynamic images and self-efficacy, whereas static images show the opposite pattern (Di Corrado et al., 2025). However, these conclusions are primarily based on limited empirical evidence. Due to the scarcity of data, the present meta-analysis was unable to evaluate whether different types of imagery practice differ significantly in their effects. Therefore, practicing imagery correctly might help enhance athletes’self-efficacy, while athletes with higher self-efficacy levels may also be more likely to engage in imagery more frequently during training.

Similarly, Callow et al. have summarized that dynamic images can enhance the vividness of imagery practice and are the best imagery strategy for improving athletes’ confidence (Callow et al., 2001, 2006). Furthermore, athletes who experience vivid dynamic images often feel more confident because this process allows them to rehearse and improve their performance (Di Corrado et al., 2020). Additionally, some evidence suggests that mastering imagery ability is the best predictor of athletes’ confidence improvement and can also alleviate their negative emotional state (Budnik-Przybylska et al., 2022; Williams and Cumming, 2012; Williams and Cumming, 2016). Hall et al. concluded further that cognitive specific and MG-M imagery were significant predictors of sport confidence in practice and competition (Hall et al., 2009). There is a positive correlation between the use of MG-M imagery and the improvement of athlete confidence (Strachan and Munroe-Chandler, 2006). Thus, appropriate types of imagery practice are necessary to improve athletes’ mental health in a targeted manner. However, due to limited data, we were unable to explore the differential effects of various types of imagery practice on athletes’ mental health in this study. Future research is warranted to specifically investigate whether different types of imagery practice produce distinct effects on athletes’ mental health.

Finally, a small number of studies have indicated that the integration of imagery practice with other psychological skills training may contribute to improvements in athletes’ confidence. Hidayat et al. concluded that the combination of imagery practice and self-talk can effectively improve the confidence of novice badminton players (Hidayat et al., 2023). Two interventions can complement each other. Imagery practice can connect the motor system and verbal system through internal representation, while self-talk generates language that activates the image motion of internal representation (Longstaff, 2011). Additionally, research indicates that the combination of imagery and relaxation yields a more substantial positive impact on athletes’ mental toughness than imagery practice alone (Bhambri et al., 2005). However, some participants during experiments expressed that combining multiple psychological skills training would increase the difficulty and complexity of the training (Weinberg et al., 1983). Therefore, despite existing evidence supporting the effectiveness of multi-component psychological skills training in promoting athletes’ mental health, our meta-analysis was unable to confirm the efficacy of combining imagery practice with other psychological strategies, largely due to inadequate sample sizes and insufficient data to support robust conclusions.

Additionally, imagery practice does not seem to be the most effective among all psychological skills training. Some evidence showed that imagery practice is less successful than relaxation techniques to improve athletes’ anxiety level (Karimian et al., 2010), and our findings also find that the effect of imagery practice on athletes’ mental health appears to be notably weaker than that of video observation. This discrepancy may be due to the inclusion of only a single study involving video-based interventions, leading to limited data support. Therefore, this was an exploratory analysis, and the findings should be interpreted with caution. Additional high-quality research is needed to further evaluate and validate the comparative effectiveness of these approaches. At present, no definitive conclusions can be drawn regarding the relative superiority of these interventions.

On the other side, the results of this study indicated a favorable effect of imagery practice exclusively among tennis players, with no statistically significant effects observed in other athlete groups. This finding is consistent with recent evidence showing that imagery practice has been used to enhance motivation, self-confidence, and emotional regulation, while reducing anxiety among tennis players (Fekih et al., 2021; Nicolas et al., 2025; Robin and Dominique, 2022; Terry et al., 1995). The positive effects of imagery practice in tennis players may be due to the sport’s high cognitive and emotional demands. Theoretical models suggest that tennis involves frequent pauses, complex visuospatial processing, and the need for emotional control, which closely align with the psychological mechanisms of imagery practice (Robin and Dominique, 2022). Moreover, research has shown that the effectiveness of internal versus external imagery may vary by skill type, such as open or closed tennis skills, supporting a task-specific approach to imagery design (Dana and Gozalzadeh, 2017). However, the lack of statistically significant results in other athlete groups may be attributed to insufficient data rather than the ineffectiveness of imagery practice itself. Similarly, the significant effects observed in tennis players should be interpreted with caution, as they are based on data from only three included studies, limiting the robustness of this finding. Therefore, although some positive evidence supports the effectiveness of imagery practice in improving athletes’ mental health, there remains a significant lack of data for specific sports and types of imagery practice. This limitation, marked by insufficient sample sizes, hinders the ability to draw robust conclusions.

### 4.3 The dose–response relationship of imagery practice on mental health

To our knowledge, this is the first multilevel meta-analysis exploring the optimal dosage of imagery practice for improving athletes’ mental health. The results showed that imagery practice for about 45 min, once a week for one hundred days may be associated with greatest positive effect on athletes’ mental health.

Prior research or reviews have proposed optimal imagery dosage primarily for performance enhancement, while neglecting the exploration of imagery dosage for mental health. For instance, Driskell et al. suggested that the optimal dosage for psychological skills training suitable for athletes is 20.8 min (Driskell et al., 1994). Furthermore, Itoh et al. have indicated that four weekly and 13–15 min each time for imagery practice have the best effect on improving the performance of basketball players (Itoh et al., 2022, 2023). The latest meta-analysis showed the optimal dose of imagery practice for muscle growth in adult populations is 4 weeks, three times a week, each lasting 15 min (Paravlic et al., 2018). It is also suggested that the more imagery practice is conducted, the more obvious the benefits for athletes (Simonsmeier et al., 2018). This supports our conclusion that long-term imagery practice is beneficial for improving athletes’ mental health.

Additionally, regarding the intensity of imagery practice, our findings suggest that a 45-min session may yield more favorable outcomes compared to 60-min sessions. However, this result may be attributed to the limited number of studies examining 60-min imagery interventions, which constrains the robustness of the available evidence. To our knowledge, our study is among the first to explore the potential optimal dosage of imagery practice for enhancing athletes’ mental health. However, given that only 24 randomized controlled trials were included, the limited sample size may affect the stability and generalizability of the conclusions. Further empirical studies with larger and more diverse samples are warranted to strengthen the robustness of these findings.

On the other hand, moderation analysis shows that both age and gender may be negatively correlated with the effect of imagery practice on mental health, which means that the older the participants, the less significant the effect of imagery practice, and the effect of imagery practice on female athletes seems to be less pronounced in female athletes than in males. However, Robin et al. concluded that in current research on imagery practice, the representation of female athletes is relatively weak (Robin and Dominique, 2022). Therefore, this study was an exploratory analysis, and the findings should be interpreted with caution. Dose–response patterns may differ by sport, gender, and age. Due to the lack of data, this conclusion is not robust, so additional research is needed to provide more robust evidence.

### 4.4 Strengths and limitations

To our knowledge, this study represents the first Bayesian multilevel meta-analysis to comprehensively synthesize the effects of imagery practice on athletes’ mental health. It is also the first to propose an optimal dosage of imagery practice tailored to enhance mental health outcomes in athletic populations. This provides further sufficient evidence for the potential application of imagery practice in the field of sports, enhances the development prospects of imagery practice in the athlete population, and increases the attention of athletes and coaches to psychological skills training.

However, there are still some limitations that need to be addressed. First, this meta-analysis included a relatively small number of randomized controlled trials, and the sample size of athletes across studies was limited. Future research should incorporate larger sample sizes and high-quality randomized controlled designs to provide more robust and reliable evidence. Secondly, although the high heterogeneity observed in the null models was substantially reduced in both the athlete model and the mental health model, it still undermines the robustness of the study’s conclusions. The heterogeneity may be attributed to variations in athlete types and mental health outcomes, thereby limiting the reliability and generalizability of the results. Thirdly, evidence of publication bias was detected in the data, which further weakens the robustness of the findings and lowers the overall quality of the evidence. To improve the reliability of future research, we emphasize the need for more rigorous experimental designs, including the proper implementation of blinding procedures during both the intervention and outcome assessment phases. Additionally, random assignment should be conducted in a fully randomized and standardized manner to ensure internal validity and minimize selection bias.

Fourthly, this study did not investigate which specific types of imagery practice (e.g., motivational-general mastery [MG-M], cognitive-specific [CS], dynamic vs. static imagery) are most effective for improving particular aspects of athletes’ mental health, such as anxiety, confidence, or self-efficacy. Future research should systematically compare the effects of different imagery techniques across diverse psychological outcomes and athlete populations (e.g., by age, sport type, or performance level) to determine the most beneficial combinations. Fifthly, although our findings did not reveal significant mental health benefits from combining imagery practice with other psychological skills training, this result may stem from the limited number of studies addressing such combinations. We encourage future research to examine whether certain psychological skills (e.g., self-talk, goal-setting, or mindfulness) synergize more effectively with imagery in promoting mental health. Finally, while it is hypothesized that integrating imagery practice with physical exercise may lead to greater mental health benefits than imagery alone, this remains an open question. We emphasize that this suggestion is speculative and requires empirical validation through well-designed experimental studies. Simultaneously, most of the research on imagery practice primarily focuses on athletes, often overlooking other demographic groups. Future studies should expand beyond athletes to investigate the effects of imagery practice on the physical capabilities and mental health of students, children, or the elderly, as more comprehensive data is needed to support these claims.

## 5 Conclusion

To our knowledge, this study is the first to employ a Bayesian multilevel framework to systematically evaluate the effects and optimal dosage of imagery practice on athletes’ mental health. Results demonstrate that imagery practice significantly may enhance mental health outcomes, particularly anxiety, confidence, and self-efficacy, with possible optimal benefits observed at a dosage of 45 min per week for 100 days. These findings extend existing psychological skills training literature by providing evidence-based dosage guidelines and highlighting differential effects by sport type and gender.

Future research should address several gaps. First, larger and more diverse RCTs are needed to validate the observed dosage-response relationships. Second, studies should explore which types of imagery practice (e.g., MG-M vs. CS imagery) are most effective for specific psychological outcomes. Third, researchers should examine whether combining imagery with other psychological techniques (e.g., self-talk, mindfulness) enhances its effectiveness. Finally, expanding the study population to include non-athlete groups such as adolescents, older adults, or clinical populations could broaden the applicability of imagery-based interventions.

Despite its strengths, this study has several limitations. First, most of the analyses (e.g., Gender, Age, Imagery types) in this study should be considered preliminary due to limited data availability. As a result, the conclusions remain unstable and require further confirmation through large-scale, high-quality experimental studies in the future. Second, moderate heterogeneity across studies may have influenced pooled estimates. Third, evidence of publication bias suggests a potential inflation of the observed effects. Fourth, variations in intervention content and delivery were not examined in detail, limiting conclusions about the most effective imagery types.

## Data Availability

Publicly available datasets were analyzed in this study. This data can be found here: https://osf.io/k2buz/?view_only=103fffebd2ba4a9bb8e428e04c902c33.

## References

[ref1] Abbas A. Hefnawy M. T. Negida A. (2024). Meta-analysis accelerator: a comprehensive tool for statistical data conversion in systematic reviews with meta-analysis. BMC Med. Res. Methodol. 24:243. doi: 10.1186/s12874-024-02356-6, PMID: 39425031 PMC11487830

[ref2] Afzali S. MolaNorouzi K. (2023). The effect of mindfulness and PETTLEP imagery on competitive state anxiety and the performance of equestrian athletes of show jumping discipline. Motor Learn. Growth J. 16, 83–99. doi: 10.22059/jsmdl.2023.364125.1745

[ref3] Alwan M. A. Zakaria H. A. A. Rahim M. R. A. Hamid N. A. Fuad M. D. F. (2013). Comparison between two relaxation methods on competitive state anxiety among college soccer teams during pre-competition stage. Int. J. Advanced Sport Sci. Res. 1, 90–104.

[ref4] Bhambri E. Dhillon P. K. Sahni S. P. (2005). Effect of psychological interventions in enhancing mental toughness dimensions of sports persons. J. Indian Acad. Appl. Psychol. 31, 65–70.

[ref5] Boetje J. Van De Schoot R. (2024). The SAFE procedure: a practical stopping heuristic for active learning-based screening in systematic reviews and meta-analyses. Syst. Rev. 13:81. doi: 10.1186/s13643-024-02502-7, PMID: 38429798 PMC10908130

[ref6] Braginsky M. Mathur M. VanderWeele T. J. (2019). PublicationBias: sensitivity analysis for publication bias in meta-analyses10.1111/rssc.12440 PMC7590147 33132447

[ref7] Budnik-Przybylska D. Huzarska I. Karasiewicz K. (2022). Does imagery ability matter for the relationship between temperament and self-confidence in team and individual sport disciplines? Front. Psychol. 13:893457. doi: 10.3389/fpsyg.2022.893457, PMID: 35898998 PMC9311684

[ref8] Bürkner P.-C. (2018). Advanced Bayesian multilevel Modeling with the R package brms. The R Journal 10:395. doi: 10.32614/RJ-2018-017

[ref9] Callow N. Hardy L. Hall C. (2001). The effects of a motivational general-mastery imagery intervention on the sport confidence of high-level badminton players. Res. Q. Exerc. Sport 72, 389–400. doi: 10.1080/02701367.2001.10608975, PMID: 11770788

[ref10] Callow N. Roberts R. Fawkes J. Z. (2006). Effects of dynamic and static imagery on vividness of imagery, skiing performance, and confidence. J. Imagery Res. Sport Physical Activity 1:Article 2. doi: 10.2202/1932-0191.1001

[ref11] Chungath A. F. Sudhesh N. T. Gupta S. Divekar S. (2022). Efficacy of a video Modeling and imagery-controlled trial intervention in a non-Western adolescent population: a case study. Case studies in sport and exercise. Psychology 6:S1-24-S1-37. doi: 10.1123/cssep.2022-0009

[ref12] Commodari E. (2024). Mental imagery in education: What impact on the relationships with visuospatial processing and school performance in junior high school students?

[ref13] Cumming J. Nordin S. M. Horton R. Reynolds S. (2006). Examining the direction of imagery and self-talk on dart-throwing performance and self efficacy. Sport Psychol. 20, 257–274. doi: 10.1123/tsp.20.3.257

[ref14] Dana A. Gozalzadeh E. (2017). Internal and external imagery effects on tennis skills among novices. Percept. Mot. Skills 124, 1022–1043. doi: 10.1177/0031512517719611, PMID: 28799864

[ref15] Dhiman C. Bedi H. S. (2010). Effect of autogenic training and mental imagery on the trait anxiety of the hockey players. Br. J. Sports Med. 44:i60.1-i60. doi: 10.1136/bjsm.2010.078725.201

[ref16] Di Corrado D. Guarnera M. Guerrera C. S. Maldonato N. M. Di Nuovo S. Castellano S. . (2020). Mental imagery skills in competitive young athletes and non-athletes. Front. Psychol. 11:633. doi: 10.3389/fpsyg.2020.00633, PMID: 32362857 PMC7180224

[ref17] Di Corrado D. Tortella P. Coco M. Guarnera M. Tusak M. Parisi M. C. (2025). Mental imagery and stress: the mediating role of self-efficacy in competitive martial arts athletes. Front. Psychol. 16:1517718. doi: 10.3389/fpsyg.2025.1517718, PMID: 40040664 PMC11876373

[ref18] Digitizer G. G. (2020). Getdata-Graph-Digitizer. Available online. (2020). Available online at: http://getdata-graph-digitizer.com/ (Accessed March 2025).

[ref19] Driskell J. E. Copper C. Moran A. (1994). Does mental practice enhance performance? J. Appl. Psychol. 79, 481–492. doi: 10.1037/0021-9010.79.4.481

[ref20] Fastame M. C. (2021). Visuo-spatial mental imagery and geometry skills in school-aged children. Sch. Psychol. Int. 42, 324–337. doi: 10.1177/0143034321992458

[ref21] Fekih S. Zguira M. S. Koubaa A. Bettaieb A. Hajji J. Bragazzi N. L. . (2021). Effects of mental training through imagery on the competitive anxiety of adolescent tennis players fasting during Ramadan: a randomized, controlled experimental study. Front. Nutr. 8:713296. doi: 10.3389/fnut.2021.713296, PMID: 34869512 PMC8633113

[ref22] Feltz D. L. Riessinger C. A. (1990). Effects of in vivo emotive imagery and performance feedback on self-efficacy and muscular endurance. J. Sport Exerc. Psychol. 12, 132–143. doi: 10.1123/jsep.12.2.132

[ref23] Gouttebarge V. Castaldelli-Maia J. M. Gorczynski P. Hainline B. Hitchcock M. E. Kerkhoffs G. M. . (2019). Occurrence of mental health symptoms and disorders in current and former elite athletes: a systematic review and meta-analysis. Br. J. Sports Med. 53, 700–706. doi: 10.1136/bjsports-2019-100671, PMID: 31097451 PMC6579497

[ref24] Grubic N. Jain S. Mihajlovic V. Thornton J. S. Johri A. M. (2021). Competing against COVID-19: have we forgotten about student-athletes’ mental health? Br. J. Sports Med. 55, 950–951. doi: 10.1136/bjsports-2021-104218, PMID: 34045286 PMC8380890

[ref25] Guarnera M. Pellerone M. Commodari E. Valenti G. D. Buccheri S. L. (2019). Mental images and school learning: a longitudinal study on children. Front. Psychol. 10:2034. doi: 10.3389/fpsyg.2019.02034, PMID: 31620040 PMC6760037

[ref26] Haddaway N. R. Page M. J. Pritchard C. C. McGuinness L. A. (2022). PRISMA 2020: an R package and shiny app for producing PRISMA 2020-compliant flow diagrams, with interactivity for optimised digital transparency and open synthesis. Campbell Syst. Rev. 18:e1230. doi: 10.1002/cl2.1230, PMID: 36911350 PMC8958186

[ref27] Hall C. R. Munroe-Chandler K. J. Cumming J. Law B. Ramsey R. Murphy L. (2009). Imagery and observational learning use and their relationship to sport confidence. J. Sports Sci. 27, 327–337. doi: 10.1080/02640410802549769, PMID: 19191065

[ref28] Hammond T. Gregg M. Hrycaiko D. Mactavish J. Leslie-Toogood A. (2012). The effects of a motivational general-mastery imagery intervention on the imagery ability and self-efficacy of inter-collegiate golfers. J. Imagery Res. Sport Physical Activity 7. doi: 10.1515/1932-0191.1066

[ref29] Harrer M. Cuijpers P. Furukawa T. A. Ebert D. D. (2021). Doing Meta-analysis with R: A hands-on guide. 1st Edn. Boca Raton, FL, USA: Chapman and Hall/CRC.

[ref30] Harris D. Wilkinson S. Ellmers T. (2023). From fear of falling to choking under pressure: a predictive processing perspective of disrupted motor control under anxiety. Neurosci. Biobehav. Rev. 148:105115. doi: 10.1016/j.neubiorev.2023.105115 36906243

[ref31] Hidayat Y. Yudiana Y. Hambali B. Sultoni K. Ustun U. D. Singnoy C. (2023). The effect of the combined self-talk and mental imagery program on the badminton motor skills and self-confidence of youth beginner student-athletes. BMC Psychol. 11:35. doi: 10.1186/s40359-023-01073-x, PMID: 36737818 PMC9898987

[ref32] Higgins J. P. T. Altman D. G. Gotzsche P. C. Juni P. Moher D. Oxman A. D. . (2011). The cochrane collaboration’s tool for assessing risk of bias in randomised trials. BMJ 343:d5928. doi: 10.1136/bmj.d5928 22008217 PMC3196245

[ref33] Holzinger A. (2016). Interactive machine learning for health informatics: when do we need the human-in-the-loop? Brain Informatics 3, 119–131. doi: 10.1007/s40708-016-0042-6, PMID: 27747607 PMC4883171

[ref34] Hut M. Minkler T. O. Glass C. R. Weppner C. H. Thomas H. M. Flannery C. B. (2023). A randomized controlled study of mindful sport performance enhancement and psychological skills training with collegiate track and field athletes. J. Appl. Sport Psychol. 35, 284–306. doi: 10.1080/10413200.2021.1989521

[ref35] Itoh S. Morris T. Spittle M. (2022). Examining the frequency variable in the imagery dose-response relationship. Asian J. Sport and Exercise Psychol. 2, 122–130. doi: 10.1016/j.ajsep.2022.06.003

[ref36] Itoh S. Morris T. Spittle M. (2023). Examining duration in the imagery dose-response relationship. J. Imagery Res. Sport Physical Activity 18:20220020. doi: 10.1515/jirspa-2022-0020

[ref37] Jeannerod M. (1994). The representing brain: neural correlates of motor intention and imagery. Behav. Brain Sci. 17, 187–202. doi: 10.1017/S0140525X00034026

[ref38] Jeannerod M. (2001). Neural simulation of action: A unifying mechanism for motor cognition. NeuroImage 14, S103–S109. doi: 10.1006/nimg.2001.0832 11373140

[ref39] Kanthack T. F. D. Bigliassi M. Vieira L. F. Altimari L. R. (2013). Efeito agudo da imagética no desempenho de lances livres e percepção de autoeficácia em atletas. Revista Brasileira Cineantropometria Desempenho Humano 16, 47–57. doi: 10.5007/1980-0037.2014v16n1p47

[ref40] Karimian M. Kashefolhagh F. Dadashi M. S. Chharbaghi Z. (2010). The effect of relaxation and mental imagery on self-efficacy, competitive anxiety and sportive performance. Br. J. Sports Med. 44:i57.2-i57. doi: 10.1136/bjsm.2010.078725.192

[ref41] Kass R. E. Raftery A. E. (1995). Bayes factors. J. Am. Stat. Assoc. 90, 773–795. doi: 10.1080/01621459.1995.10476572

[ref42] Kruschke J. K. Liddell T. M. (2018). The Bayesian new statistics: hypothesis testing, estimation, meta-analysis, and power analysis from a Bayesian perspective. Psychon. Bull. Rev. 25, 178–206. doi: 10.3758/s13423-016-1221-4, PMID: 28176294

[ref43] Lee Howard W. Reardon J. P. (1986). Changes in the self concept and athletic performance of weight lifters through a cognitive-hypnotic approach: an empirical study. Am. J. Clin. Hypn. 28, 248–257. doi: 10.1080/00029157.1986.10402661, PMID: 3717057

[ref44] Li Z. Moritz S. E. Liu H. (2024). Motor imagery as a potential tool to alleviate choking under pressure in sports performance. J. Imagery Res. Sport Physical Activity 19:20240012. doi: 10.1515/jirspa-2024-0012

[ref45] Longstaff L. (2011). Factors affecting the optimal use of imagery and self talk in golfers: University of Northumbria at Newcastle.

[ref46] Makowski D. Ben-Shachar M. Lüdecke D. (2019). bayestestR: describing effects and their uncertainty, existence and significance within the Bayesian framework. J. Open Source Softw. 4:1541. doi: 10.21105/joss.01541

[ref47] Marshall E. A. Gibson A.-M. (2017). The effect of an imagery training intervention on self-confidence, anxiety and performance in acrobatic gymnastics – a pilot study. J. Imagery Res. Sport and Physical Activity 12:20160009. doi: 10.1515/jirspa-2016-0009

[ref48] Martin K. A. Hall C. R. (1995). Using mental imagery to enhance intrinsic motivation. J. Sport Exerc. Psychol. 17, 54–69. doi: 10.1123/jsep.17.1.54

[ref49] Mathur M. B. VanderWeele T. J. (2020). Sensitivity analysis for publication bias in meta-analyses. J. R. Stat. Soc. Ser. C Appl. Stat. 69, 1091–1119. doi: 10.1111/rssc.12440, PMID: 33132447 PMC7590147

[ref50] McAlister A. Cutler D. (2024). A pilot feasibility study comparing mindfulness and imagery interventions on sport anxiety in division 1 volleyball players. J. Sport Behav. 47, 59–65.

[ref51] McGuinness L. A. Higgins J. P. T. (2021). Risk-of-bias VISualization (robvis): an R package and shiny web app for visualizing risk-of-bias assessments. Res. Synth. Methods 12, 55–61. doi: 10.1002/jrsm.1411, PMID: 32336025

[ref52] Mesagno C. Beckmann J. (2017). Choking under pressure: theoretical models and interventions. Curr. Opin. Psychol. 16, 170–175. doi: 10.1016/j.copsyc.2017.05.015, PMID: 28813345

[ref53] Mguidich H. Zoudji B. Khacharem A. (2024). Which modality is best for delivering an imagery script? Evidence for desirable difficulty effect. Int. J. Sport Exerc. Psychol. 1–19, 1–19. doi: 10.1080/1612197X.2024.2345704, PMID: 40640861

[ref54] Monsma E. Mensch J. Farroll J. (2009). Keeping your head in the game: sport-specific imagery and anxiety among injured athletes. J. Athl. Train. 44, 410–417. doi: 10.4085/1062-6050-44.4.410, PMID: 19593424 PMC2707070

[ref55] Munroe-Chandler K. J. Hall C. R. Fishburne G. J. Shannon V. (2005). Using cognitive general imagery to improve soccer strategies. Eur. J. Sport Sci. 5, 41–49. doi: 10.1080/17461390500076592

[ref56] Munzert J. Lorey B. Zentgraf K. (2009). Cognitive motor processes: the role of motor imagery in the study of motor representations. Brain Res. Rev. 60, 306–326. doi: 10.1016/j.brainresrev.2008.12.024, PMID: 19167426

[ref57] National Collegiate Athletic Association. (2022). NCAA Student-Athlete COVID-19 Well-being Study: Survey Results—December 2021. Available online at: http://www.ncaa.org/about/resources/research/ncaa-student-athlete-covid-19-well-being-study

[ref58] Nicolas R. Carien R. Ouarti Y. Laurent D. (2025). Beneficial effects of imagination of successful action after an actual error on baseline performances in non-expert young tennis players. Psychol. Res. 89:23. doi: 10.1007/s00426-024-02051-7, PMID: 39549137 PMC11568982

[ref59] Noh Y.-E. Morris T. Andersen M. B. (2007). Psychological intervention programs for reduction of injury in ballet dancers. Res. Sports Med. 15, 13–32. doi: 10.1080/15438620600987064 17365949

[ref60] Page S. J. Sime W. Nordell K. (1999). The effects of imagery on female college swimmers’ perceptions of anxiety. The Sport Psychologist 13, 458–469. doi: 10.1123/tsp.13.4.458

[ref61] Paravlic A. H. Slimani M. Tod D. Marusic U. Milanovic Z. Pisot R. (2018). Effects and dose–response relationships of motor imagery practice on strength development in healthy adult populations: a systematic review and meta-analysis. Sports Med. 48, 1165–1187. doi: 10.1007/s40279-018-0874-8 29541965

[ref62] Raboin S. (2024). Visualizing victory: the role of imagery in empowering athletes battling obsessive-compulsive disorder. J. Imagery Res. Sport Physical Activity 19:20240006. doi: 10.1515/jirspa-2024-0006

[ref63] Robin N. Dominique L. (2022). Mental imagery and tennis: a review, applied recommendations and new research directions. Movement Sport Sci. Motricité. 127, 57–75. doi: 10.1051/sm/2022009

[ref64] Röver C. (2020). Bayesian random-effects Meta-analysis using the bayesmeta R package. J. Stat. Softw. 93, 1–51. doi: 10.18637/jss.v093.i06

[ref65] Rumeau V. Grospretre S. Babault N. (2023). Post-activation performance enhancement and motor imagery are efficient to emphasize the effects of a standardized warm-up on Sprint-running performances. Sports 11:108. doi: 10.3390/sports11050108, PMID: 37234064 PMC10221695

[ref66] Ryan R. (2016). How to GRADE the quality of the evidence. Cochrane Consumers and Communication Group. Available online at: http://cccrg.cochrane.org/author-resources (Accessed April 2025).

[ref67] Simonsmeier B. A. Frank C. Gubelmann H. Schneider M. (2018). The effects of motor imagery training on performance and mental representation of 7- to 15-year-old gymnasts of different levels of expertise. Sport Exerc. Perform. Psychol. 7, 155–168. doi: 10.1037/spy0000117

[ref68] Strachan L. Munroe-Chandler K. (2006). Using imagery to predict self-confidence and anxiety in young elite athletes. J. Imagery Res. Sport Physical Activity. 1, 1–19. doi: 10.2202/1932-0191.1004

[ref69] Taylor J. A. Shaw D. F. (2002). The effects of outcome imagery on golf-putting performance. J. Sports Sci. 20, 607–613. doi: 10.1080/026404102320183167, PMID: 12190280

[ref70] Terry P. Coakley L. Karageorghis C. (1995). Effects of intervention upon Precompetition state anxiety in elite junior tennis players: the relevance of the matching hypothesis. Percept. Mot. Skills 81, 287–296. doi: 10.2466/pms.1995.81.1.287, PMID: 8532469

[ref71] Toth A. J. McNeill E. Hayes K. Moran A. P. Campbell M. (2020). Does mental practice still enhance performance? A 24 year follow-up and meta-analytic replication and extension. Psychol. Sport Exerc. 48:101672. doi: 10.1016/j.psychsport.2020.101672

[ref72] van de Schoot R. de Bruin J. Schram R. Zahedi P. de Boer J. Weijdema F. . (2021). An open source machine learning framework for efficient and transparent systematic reviews. 3, 125–133. doi: 10.1038/s42256-020-00287-7

[ref73] Veritas Health Innovation (2023). Covidence systematic review software [Computer software]. Available online at: www.covidence.org (Accessed March 2025).

[ref74] Volgemute K. Vazne Ž. Krauksta D. (2021). The relationship between imagery and physical self-efficacy in athletes. LASE J. Sport Sci. 12, 3–11.

[ref75] Volgemute K. Vazne Z. Krauksta D. (2024). An intervention into imagery and self-efficacy: enhancing athletic achievements of alpine skiers. Educ. Sci 14:513. doi: 10.3390/educsci14050513

[ref76] Wang Z. Nayfeh T. Tetzlaff J. O’Blenis P. Murad M. H. (2020). Error rates of human reviewers during abstract screening in systematic reviews. PLoS One 15:e0227742. doi: 10.1371/journal.pone.0227742, PMID: 31935267 PMC6959565

[ref77] Weinberg R. S. Chan R. Jackson A. (1983). Mental preparation strategies and performance: is a combination of techniques better than a single technique? J. Sports Sci. 1, 211–216. doi: 10.1080/02640418308729682

[ref78] Williams S. E. Cumming J. (2012). Sport imagery ability predicts trait confidence, and challenge andthreat appraisal tendencies. Eur. J. Sport Sci. 12, 499–508. doi: 10.1080/17461391.2011.630102

[ref79] Williams S. E. Cumming J. (2016). Athlete imagery ability: a predictor of confidence and anxiety intensity and direction. Int. J. Sport Exerc. Psychol. 14, 268–280. doi: 10.1080/1612197X.2015.1025809

[ref80] Wirbiezcas M. (2019). The mediation of affect on imagery use and self-efficacy in collegiate athletes. Georgia Southern University.

[ref81] Zandi H. G. Masomi H. (2010). The effects of imagery in soccer players perceptions of anxiety during penalty kick. Br. J. Sports Med. 44:i61.1-i61. doi: 10.1136/bjsm.2010.078725.205

[ref82] Zervas Y. Kakkos V. (1991). Visuomotor behavior rehearsal in archery shooting performance. Percept. Mot. Skills 73, 1183–1190. doi: 10.2466/pms.1991.73.3f.1183

