# Supplementary material for: Optimal dosage and effectiveness of imagery practice on athletes’ mental health: a Bayesian multilevel meta-analysis
Source: Front Psychol. 2025 Aug 8;16:1618617. doi: 10.3389/fpsyg.2025.1618617 (PMC12372340; doi:10.3389/fpsyg.2025.1618617)
Supplement: Supplementary file 1 [file Data_Sheet_1.zip › Supplementary File/Supplementary file S1 Search strategy.docx]

**Supplementary File S1 : Search Strategy**

| EBSCO (MEDLINE complete, MEDLINE, CINAHL, PsycInfo, SPORTDiscus) Febrary 24 2025 | |
| --- | --- |
| 1 | TI(Imagery* OR “Imagery practice”) |
| 2 | AB(Imagery* OR “Imagery practice”) |
| 3 | TI(Imaging*) |
| 4 | AB(Imaging*) |
| 5 | 1 OR 2 OR 3 OR 4 |
| 6 | TX(“mental health” OR happ* OR anxiety* OR depression* OR stress* OR “mental toughness” OR motivation* OR happiness* OR “psychological well-being” OR “mental well-being” OR flow* OR pain* OR panic* OR anger* OR vividness* OR mood* OR emotion* OR concentration* OR HRV* OR cortisol*) |
| 7 | TX(athletes* OR players* OR “para-athletes” OR cyclists* OR runners* OR golfers* OR wrestlers* OR “rugby players” OR “tennis players” OR boxers* OR “disabled athletes” OR shooters*) |
| 9 | 5 AND 6 AND 7 |

| Pubmed Febrary 24 2025 | |
| --- | --- |
| 1 | (((Imagery[MeSH Terms]) OR (Imagery[Title/Abstract])) OR (Imaging[Title/Abstract])) OR ("Imagery practice"[Title/Abstract]) |
| 2 | (((((((("mental health"[MeSH Terms]) OR ("mental health"[Text Word])) OR (anxiety[Text Word])) OR (mood[Text Word])) OR (emotion[Text Word])) OR ("mental well-being"[Text Word])) OR ("psychological well-being"[Text Word])) OR ("HRV"[Text Word])) OR ("mental toughness"[Text Word]) |
| 3 | (((((((((((((athletes[MeSH Terms]) OR (athletes[Title/Abstract])) OR (athletes[Text Word])) OR (players[Text Word])) OR (players[Title/Abstract])) OR (runners[Text Word])) OR (cyclists[Text Word])) OR (boxers[Text Word])) OR (shooters[Text Word])) OR (wrestlers[Text Word])) OR (golfers[Text Word])) OR ("para-athletes"[Text Word])) OR ("rugby players"[Text Word])) OR ("tennis players"[Text Word]) |
| 4 | 1 AND 2 AND 3 |

| Web of Science Febrary 24 2025 | |
| --- | --- |
| 1 | ((TS=(Imagery* OR Image* OR "Imagery practice" OR Imaging* OR "Imagery training")) OR TI=(Imagery* OR Image* OR "Imagery practice" OR Imaging* OR "Imagery training" )) OR AB=(Imagery* OR Image* OR "Imagery practice" OR Imaging* OR "Imagery training") |
| 2 | ALL=( “mental health” OR happ* OR anxiety* OR depression* OR stress* OR “mental toughness” OR motivation* OR happiness* OR “psychological well-being” OR “mental well-being” OR flow* OR pain* OR panic* OR anger* OR vividness* OR mood* OR emotion* OR concentration* OR HRV* OR cortisol*) |
| 3 | ((ALL=(athletes* OR players* OR “para-athletes” OR cyclists* OR runners* OR golfers* OR wrestlers* OR “rugby players” OR “tennis players” OR boxers* OR “disabled athletes” OR shooters*)) OR TI=(athletes* OR players* OR “para-athletes” OR cyclists* OR runners* OR golfers* OR wrestlers* OR “rugby players” OR “tennis players” OR boxers* OR “disabled athletes” OR shooters*)) OR AB=(athletes* OR players* OR “para-athletes” OR cyclists* OR runners* OR golfers* OR wrestlers* OR “rugby players” OR “tennis players” OR boxers* OR “disabled athletes” OR shooters*) |
| 4 | #1 AND #2 AND #3 |
