# Supplementary material for: Optimal dosage and effectiveness of imagery practice on athletes’ mental health: a Bayesian multilevel meta-analysis
Source: Front Psychol. 2025 Aug 8;16:1618617. doi: 10.3389/fpsyg.2025.1618617 (PMC12372340; doi:10.3389/fpsyg.2025.1618617)
Supplement: Supplementary file 1 [file Data_Sheet_1.zip › Supplementary File/Supplementary file S3 Risk_of_Bias_in_Individual_Studies.docx]

Table 1: <i> Supplement File. Risk of Bias in Individual Studies </i>

| Author | Sequence generation | Allocation concealment | Blinding participants | Blinding personnel | Blinding outcome assessment | Incomplete outcome | Selective reporting | Other bias | Overall bias |
| --- | --- | --- | --- | --- | --- | --- | --- | --- | --- |
| Alwan | Low | Unclear | Unclear | Unclear | Unclear | Unclear | Low | Low | High |
| Carter | Low | Unclear | Unclear | Unclear | Unclear | Low | Low | Low | Unclear |
| Chungath | Low | Unclear | Unclear | Unclear | Unclear | Low | Low | Low | Unclear |
| Fazel | Low | Unclear | Unclear | Unclear | Unclear | Low | Low | Low | Unclear |
| Fekih 2021 | Low | Unclear | Unclear | Unclear | Unclear | Low | Low | Low | Unclear |
| Graham | Low | Low | Low | Low | Unclear | Low | Low | Low | Low |
| Hidayat | Low | Low | Low | Unclear | Unclear | Low | Low | Low | Low |
| Howard | Low | Unclear | Unclear | Unclear | Unclear | Low | Low | Low | Unclear |
| Hut | Low | Unclear | Low | Low | Unclear | High | Low | Low | High |
| Kanthack | Low | Unclear | Unclear | Unclear | Unclear | Low | Low | Low | Unclear |
| Korim | Low | Unclear | Unclear | Unclear | Unclear | Low | Low | Low | Unclear |
| Marshall | High | Unclear | Unclear | Unclear | Unclear | Low | Low | Low | High |
| McAlister | Low | Low | Low | Low | Unclear | Unclear | Low | Low | Low |
| Mguidich | Low | Unclear | Unclear | Unclear | Unclear | Low | Low | Low | Unclear |
| Nicolas | Low | Unclear | Unclear | Unclear | Unclear | Low | Low | Low | Unclear |
| Noh | High | Low | Low | Low | Low | Low | Low | Low | High |
| Page | Low | Unclear | Unclear | Unclear | Unclear | Low | Low | Low | Unclear |
| Ramsey | Low | Unclear | Unclear | Unclear | Unclear | Low | Low | Low | Unclear |
| Rhodes | Low | Unclear | Unclear | Unclear | Unclear | Low | Low | Low | Unclear |
| Rumeau | Low | Unclear | Low | Low | Unclear | Low | Low | Low | Low |
| Taylor | Low | Unclear | Unclear | Unclear | Unclear | Low | Low | Low | Unclear |
| Terry | Low | Unclear | Unclear | Unclear | Unclear | Low | Low | Low | Unclear |
| Veskovic | Low | Unclear | Unclear | Unclear | Unclear | Low | Low | Low | Unclear |
| Yahya | Low | Low | Low | Low | Unclear | Low | Low | Low | Low |
