# Supplementary material for: Optimal dosage and effectiveness of imagery practice on athletes’ mental health: a Bayesian multilevel meta-analysis
Source: Front Psychol. 2025 Aug 8;16:1618617. doi: 10.3389/fpsyg.2025.1618617 (PMC12372340; doi:10.3389/fpsyg.2025.1618617)
Supplement: Supplementary file 1 [file Data_Sheet_1.zip › Supplementary File/Supplementary file S6 The Linear Regression Plots and R2 Density Plots.docx]

**Supplementary file S6:** The Linear Regression Plots and R^2^ Density Plots


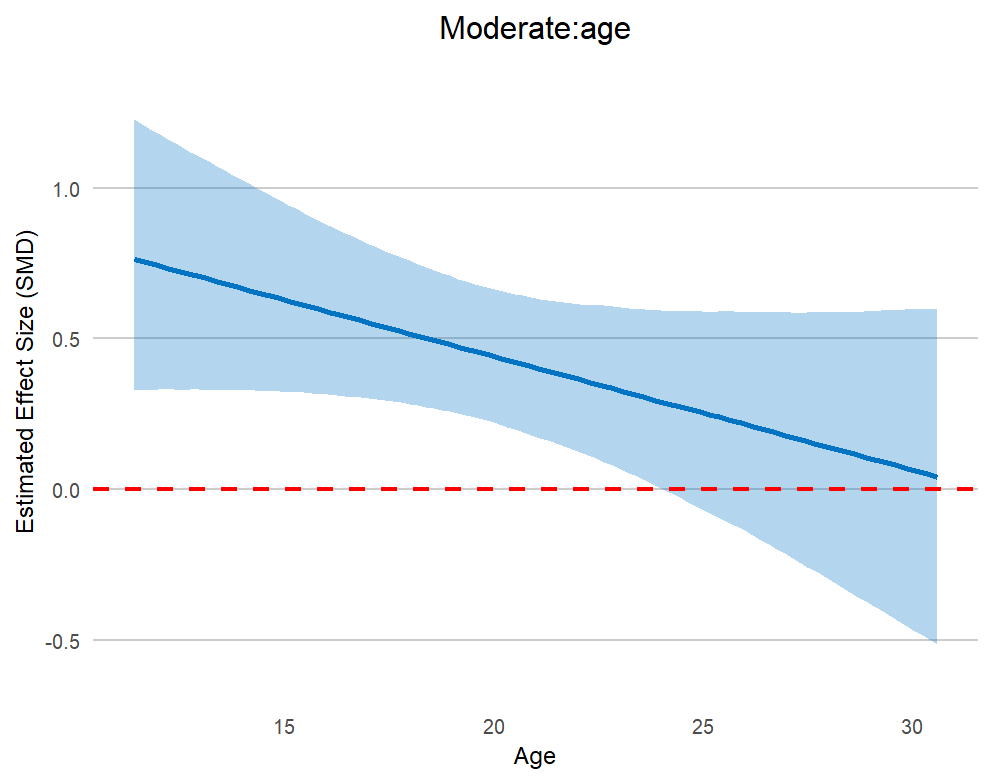


**Figure S1** The Regression Plots for Mental health (Age)


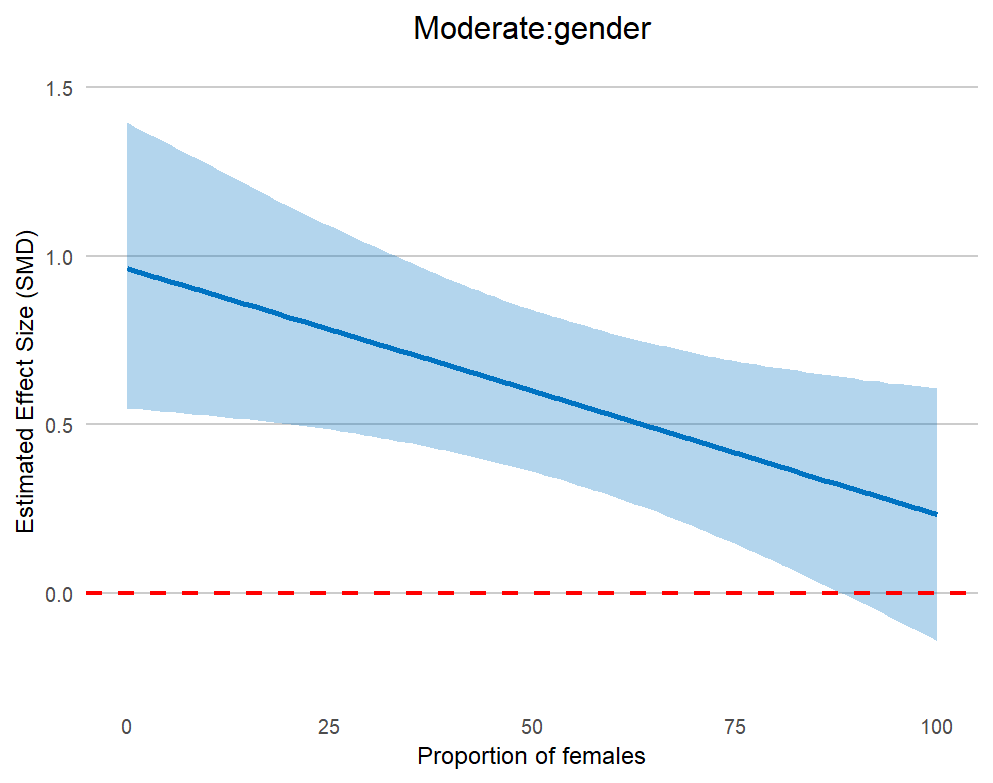


**Figure S2** The Regression Plots for Mental health (Gender)


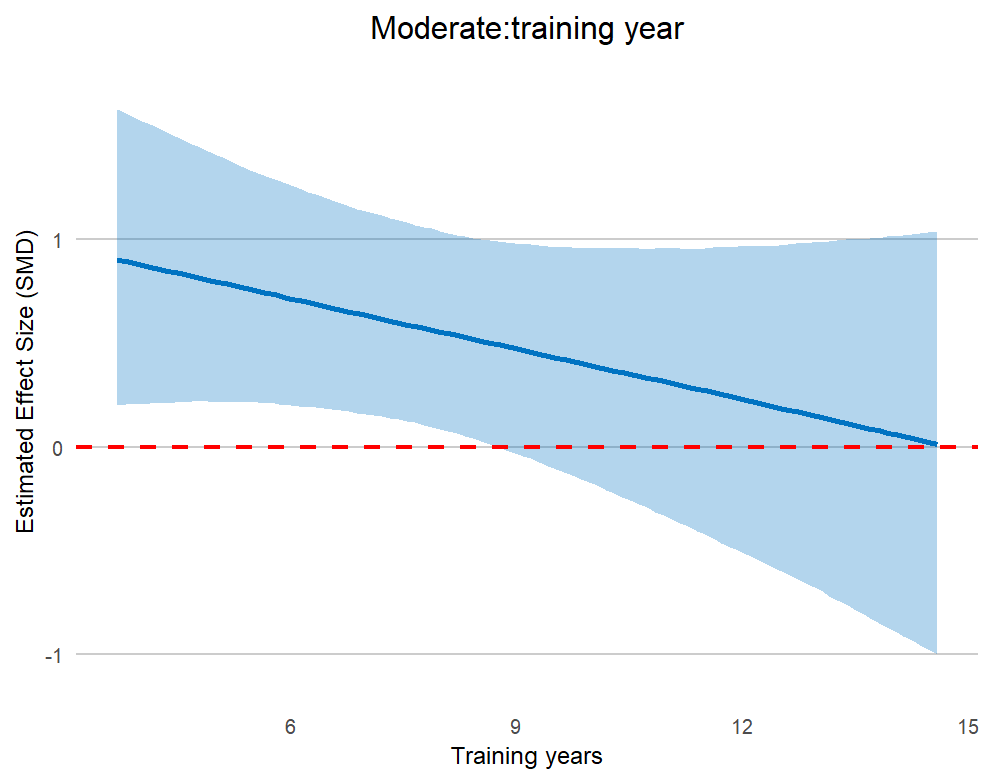


**Figure S3** The Regression Plots for Mental health (Training Years)


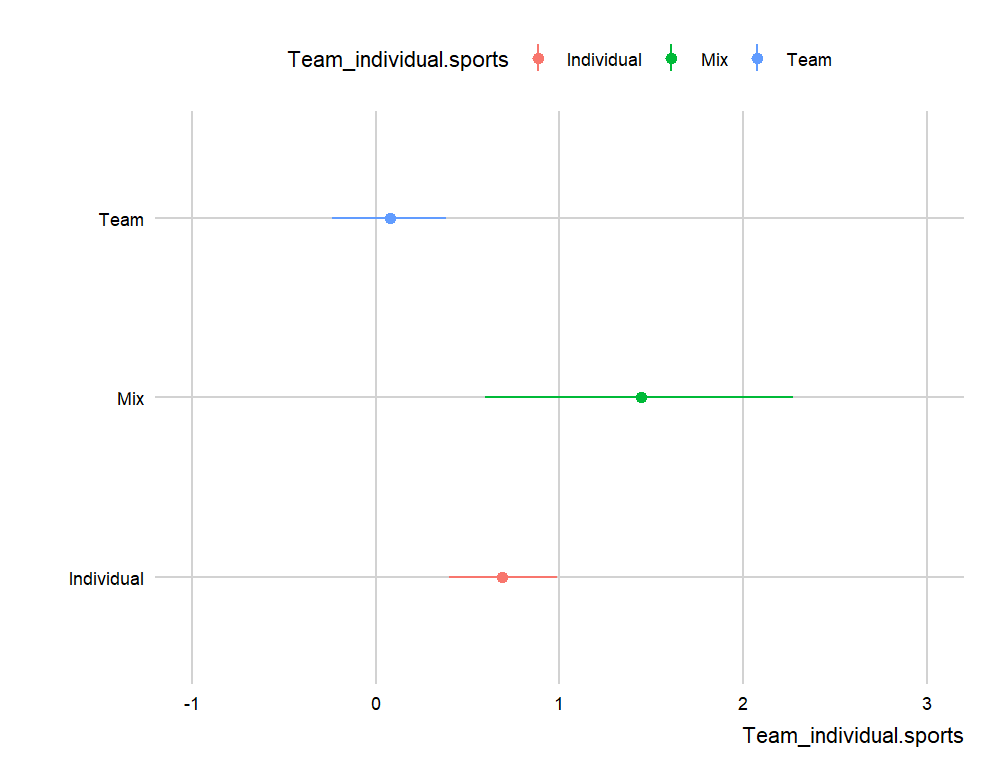


**Figure S4** The Regression Plots for Mental health (TI)


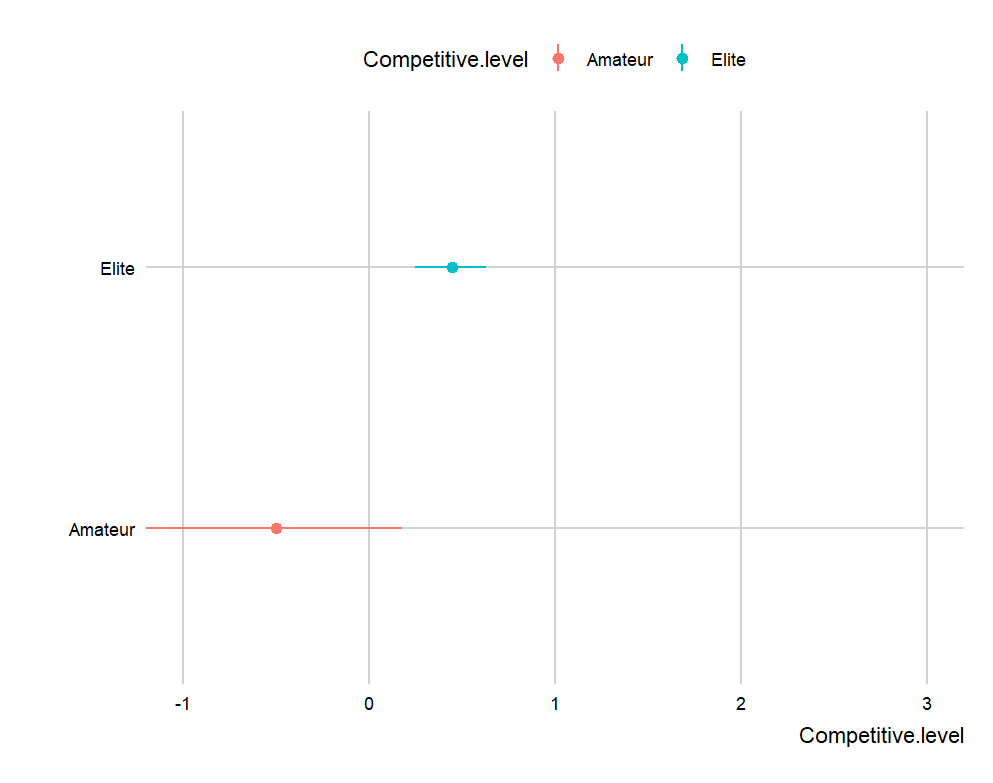


**Figure S5** The Regression Plots for Mental health (Competitive Level)


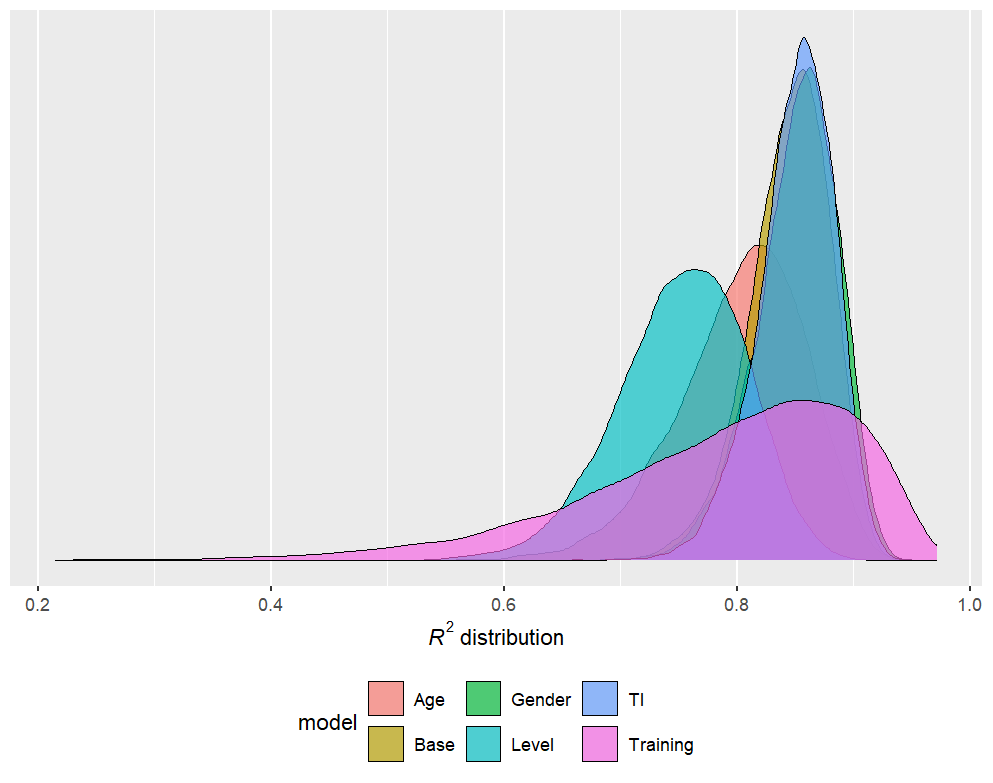


**Figure S6** The R^2^ Density Plot for Moderation Analysis (Athlete Characteristics)


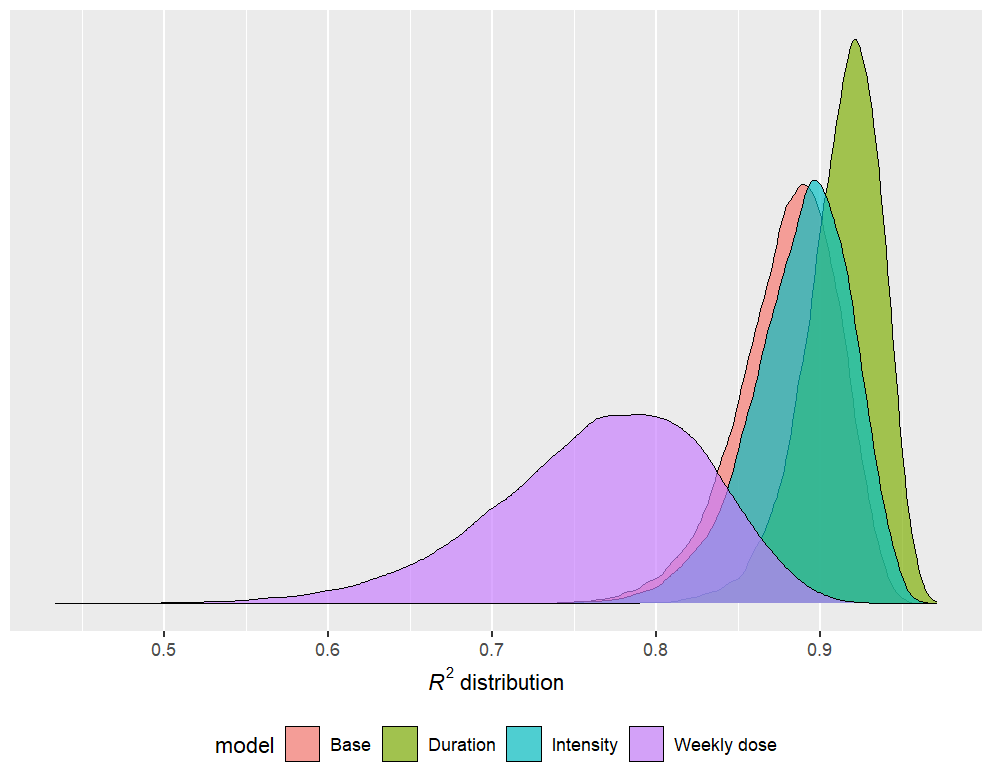


**Figure S7** The R^2^ Density Plot for Moderation Analysis (Dosage)
