# Supplementary material for: Optimal dosage and effectiveness of imagery practice on athletes’ mental health: a Bayesian multilevel meta-analysis
Source: Front Psychol. 2025 Aug 8;16:1618617. doi: 10.3389/fpsyg.2025.1618617 (PMC12372340; doi:10.3389/fpsyg.2025.1618617)
Supplement: Supplementary file 1 [file Data_Sheet_1.zip › Supplementary File/Supplementary file S8 List of Studies Included in the Meta-Analysis.docx]

**Supplementary file S16: List of Studies Included in the Meta-Analysis**

1. Noh YE, Morris T, Andersen MB. Psychological Intervention Programs for Reduction of Injury in Ballet Dancers. *Research in Sports Medicine*. 2007;15(1):13-32. doi:10.1080/15438620600987064

2. Marshall EA, Gibson AM. The Effect of an Imagery Training Intervention on Self-confidence, Anxiety and Performance in Acrobatic Gymnastics – A Pilot Study. *Journal of Imagery Research in Sport and Physical Activity*. 2017;12(1):20160009. doi:10.1515/jirspa-2016-0009

3. Taylor JA, Shaw DF. The effects of outcome imagery on golf-putting performance. *Journal of Sports Sciences*. 2002;20(8):607-613. doi:10.1080/026404102320183167

4. Nicolas R, Carien R, Ouarti Y, Laurent D. Beneficial effects of imagination of successful action after an actual error on baseline performances in non-expert young tennis players. *Psychological Research*. 2025;89(1):23. doi:10.1007/s00426-024-02051-7

5. McAlister A, Cutler D. A Pilot Feasibility Study Comparing Mindfulness and Imagery Interventions on Sport Anxiety in Division 1 Volleyball Players. *Journal of Sport Behavior*. 2024;47(4):59-65.

6. Chungath AF, Sudhesh NT, Gupta S, Divekar S. Efficacy of a Video Modeling and Imagery-Controlled Trial Intervention in a Non-Western Adolescent Population: A Case Study. *Case Studies in Sport and Exercise Psychology*. 2022;6(S1):S1-24-S1-37. doi:10.1123/cssep.2022-0009

7. Fazel F, Morris T, Watt A, Maher R. The effects of different types of imagery delivery on basketball free-throw shooting performance and self-efficacy. *Psychology of Sport and Exercise*. 2018;39:29-37. doi:10.1016/j.psychsport.2018.07.006

8. Jennifer E. C. Using Traditional and Paradoxical Imagery Interventions with Reactant Intramural Athletes. *The Sport Psychologist*. 1997;11:175-189.

9. Rumeau V, Grospretre S, Babault N. Post-Activation Performance Enhancement and Motor Imagery Are Efficient to Emphasize the Effects of a Standardized Warm-Up on Sprint-Running Performances. *Sports*. 2023;11(5):108. doi:10.3390/sports11050108

10. Graham LB. Effect of EMDR on Anxiety and Swim Times. *Journal of swimming research*. 2007;17:1-9.

11. Mguidich H, Zoudji B, Khacharem A. Which modality is best for delivering an imagery script? Evidence for desirable difficulty effect. *International Journal of Sport and Exercise Psychology*. Published online April 27, 2024:1-19. doi:10.1080/1612197X.2024.2345704

12. Kanthack TFD, Bigliassi M, Vieira LF, Altimari LR. Efeito agudo da imagética no desempenho de lances livres e percepção de autoeficácia em atletas. *Rev Bras Cineantropom Desempenho Hum*. 2013;16(1):47-57. doi:10.5007/1980-0037.2014v16n1p47

13. Korim V, Strnádelová B. The effect of imagery on performance and level of self-criticism of athletes. *Journal of Imagery Research in Sport and Physical Activity*. 2023;18(1):20220017. doi:10.1515/jirspa-2022-0017

14. Fekih S, Zguira MS, Koubaa A, et al. Effects of Mental Training Through Imagery on the Competitive Anxiety of Adolescent Tennis Players Fasting During Ramadan: A Randomized, Controlled Experimental Study. *Front Nutr*. 2021;8:713296. doi:10.3389/fnut.2021.713296

15. Vesković A, Koropanovski N, Dopsaj M, Jovanović S. EFFECTS OF A PSYCHOLOGICAL SKILL TRAINING PROGRAM ON ANXIETY LEVELS IN TOP KARATE ATHLETES. *Rev Bras Med Esporte*. 2019;25(5):418-422. doi:10.1590/1517-869220192505173969

16. Ramsey R. Examing the emotion aspect of PETTLEP-based imagery with penalty taking in soccer. *Journal of Sport Behavior*. 2010;33.

17. Hidayat Y, Yudiana Y, Hambali B, Sultoni K, Ustun UD, Singnoy C. The effect of the combined self-talk and mental imagery program on the badminton motor skills and self-confidence of youth beginner student-athletes. *BMC Psychol*. 2023;11(1):35. doi:10.1186/s40359-023-01073-x

18. Rhodes J, May J, Andrade J, Kavanagh D. Enhancing Grit Through Functional Imagery Training in Professional Soccer. *The Sport Psychologist*. 2018;32(3):220-225. doi:10.1123/tsp.2017-0093

19. Alwan MA, Zakaria HAA, Rahim MRA, Hamid NA, Fuad MDF. Comparison between Two Relaxation Methods On Competitive State Anxiety Among College Soccer Teams During Pre-Competition Stage. *International Journal of Advanced Sport Sciences Research*. 2013;1(1):90-104.

20. Hut M, Minkler TO, Glass CR, Weppner CH, Thomas HM, Flannery CB. A randomized controlled study of mindful sport performance enhancement and psychological skills training with collegiate track and field athletes. *Journal of Applied Sport Psychology*. 2023;35(2):284-306. doi:10.1080/10413200.2021.1989521

21. Lee Howard W, Reardon JP. Changes in the Self Concept and Athletic Performance of Weight Lifters Through a Cognitive-Hypnotic Approach: An Empirical Study. *American Journal of Clinical Hypnosis*. 1986;28(4):248-257. doi:10.1080/00029157.1986.10402661

22. Page SJ, Sime W, Nordell K. The Effects of Imagery on Female College Swimmers’ Perceptions of Anxiety. *The Sport Psychologist*. 1999;13(4):458-469. doi:10.1123/tsp.13.4.458

23. Terry P, Coakley L, Karageorghis C. Effects of Intervention upon Precompetition State Anxiety in Elite Junior Tennis Players: The Relevance of the Matching Hypothesis. *Percept Mot Skills*. 1995;81(1):287-296. doi:10.2466/pms.1995.81.1.287

24. Yahya MF. The Idea of Using Practice in Mind Training Program for Rugby Players to Improve Anxiety and Kicking. *International Journal of Sports Science*. 2016;6:70-75.
