# Supplementary figures and images for: Optimal dosage and effectiveness of imagery practice on athletes’ mental health: a Bayesian multilevel meta-analysis
Source: Front Psychol. 2025 Aug 8;16:1618617. doi: 10.3389/fpsyg.2025.1618617 (PMC12372340; doi:10.3389/fpsyg.2025.1618617)

**Supplementary file S7:** Graphical Abstract


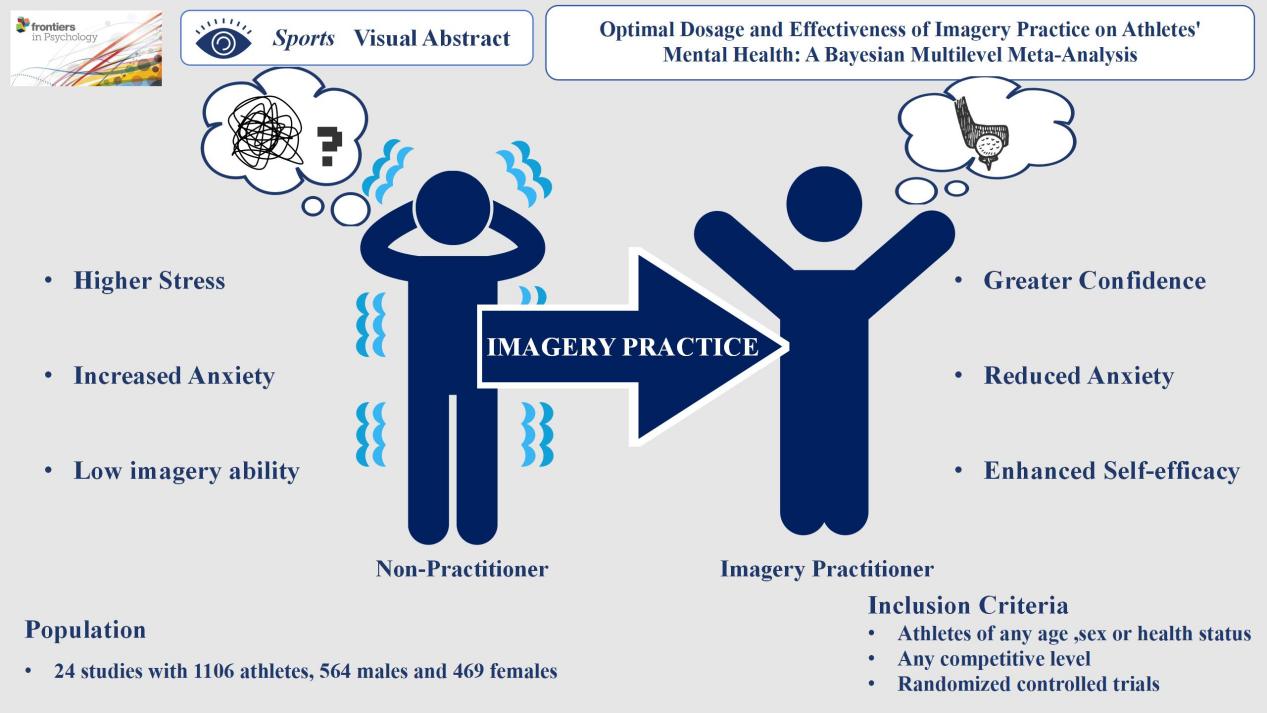


**Figure S1** Graphical Abstract

Supplement: Supplementary file 1 [file Data_Sheet_1.zip › Supplementary File/Supplementary file S7 Graphical Abstract.docx]
